# Superfast Gelation of Spider Silk-Based Artificial Silk Protein

**DOI:** 10.3390/gels10010069

**Published:** 2024-01-17

**Authors:** Fan Wen, Yu Wang, Bowen Tu, Lun Cui

**Affiliations:** 1 CCZU-JITRI Joint Bio-X Lab, School of Pharmacy & School of Biological and Food Engineering, Changzhou University, Changzhou 213164, China; wenfan25@outlook.com (F.W.); wyu30086@gmail.com (Y.W.); 2 Pathogenic Biological Laboratory, Changzhou Disease Control and Prevention Centre, Changzhou Medical Centre, Nanjing Medical University, Changzhou 213000, China; tbwchangzhou@163.com; 3 Biomaterials Lab, Changzhou AiRiBio Healthcare Co., Ltd., Changzhou 213164, China

**Keywords:** spider silk proteins, rapid gelation, hydrogel formation, self-assembly, recombinant protein

## Abstract

Spider silk proteins (spidroins) have garnered attention in biomaterials research due to their ability to self-assemble into hydrogels. However, reported spidroin hydrogels require high protein concentration and prolonged gelation time. Our study engineered an artificial spidroin that exhibits unprecedented rapid self-assembly into hydrogels at physiologically relevant conditions, achieving gelation at a low concentration of 6 mg/mL at 37 °C without external additives. Remarkably, at a 30 mg/mL concentration, our engineered protein forms hydrogels within 30 s, a feature we termed “superfast gelation”. This rapid formation is modulated by ions, pH, and temperature, offering versatility in biomedical applications. The hydrogel’s capacity to encapsulate proteins and support *E. coli* growth while inducing RFP expression provides a novel platform for drug delivery and bioengineering applications. Our findings introduce a superfast, highly adaptable, and cytocompatible hydrogel that self-assembles under mild conditions, underscoring the practical implication of rapid gelation in biomedical research and clinical applications.

## 1. Introduction

Hydrogels are a class of water-encapsulated three-dimensional (3D) polymer networks with high water content, tunable mechanical properties, biocompatibility, and the ability to respond to environmental stimuli [1,2,3]. They have long been recognized as a versatile class of materials, distinguished by their unique ability to encapsulate substantial amounts of water within a 3D matrix. The inherent properties of hydrogels are majorly dictated by their material composition, cross-linking methodologies, and fabrication techniques [4,5]. Typical materials utilized for hydrogel formation include polyethylene glycol (PEG), gelatin, collagen, chemical polyols, and silk fibroin. Cross-linking methods are pivotal in determining hydrogels’ overall strength and durability. For instance, while physical cross-linking approaches, such as hydrophobic interactions, often yield hydrogels with limited mechanical robustness, covalent strategies, like free radical polymerization, can produce hydrogels with enhanced mechanical attributes [6]. Notably, some hydrogels, especially those derived from silk fibroin, undergo self-assembly, further diversifying their structural and functional potential [7]. Fabrication methods, particularly in situ gelation and micropatterning, are crucial in dictating hydrogels’ properties and broad applications [8,9]. Micropatterning, which involves creating defined and orderly microstructures on the hydrogel surface, enables unparalleled precision in controlling cell attachment sites. On the other hand, in situ gelation is a method that facilitates the transition of a liquid precursor solution into a solid or semi-solid hydrogel directly at the desired site, making it a preferred choice for applications, like subcutaneous injections, where minimal invasiveness is crucial.

Due to these fabrication methodologies and their unique properties, hydrogels have been extensively employed in medical and biomedical research [6,10,11]. Certain hydrogels, such as those based on hyaluronic acid, have been commercialized and are widely used as fillers in cosmetic surgery [12]. In biomedical research, hydrogels have emerged as a versatile and promising tool with applications spanning various areas, from in-depth studies of physiological and pathological mechanisms to innovative approaches in tissue regeneration and targeted disease therapies. One of the hallmark features of hydrogels is their inner structure, which strikingly resembles the extracellular matrices found within living tissues. This inherent similarity makes hydrogels exceptionally suited for cell encapsulation, enabling the formation of organoids [13]. These three-dimensional miniature organs are revolutionary, offering a platform to replicate and study various diseases in a controlled environment. 

Despite the enormous promise hydrogels hold for the future of medicine and biomedical research, several challenges persist, acting as roadblocks to their optimal utilization in biomedical applications. One of the most pressing concerns is the limited availability of proteins optimized for hydrogel formation [14]. Commonly used proteins include collagen, gelatin, and fibrin, each with different mechanical strength, biocompatibility, or stability limitations. Spider silk proteins, in contrast, offer a unique combination of high tensile strength, elasticity, and biocompatibility, making them highly attractive for hydrogel development. Natural spider silk proteins are substantial structural proteins, often encompassing more than 6000 amino acid residues. Their intriguing modular design, characterized by a lengthy central repetitive domain flanked by unique globular N-terminal (NT) and C-terminal (CT) domains [15], has caught the scientific community’s attention. Recent studies, particularly by Tina Arndt and colleagues [16], have shown the hydrogel formation potential of recombinant spider silk proteins. Their groundbreaking research demonstrated that a recombinant mini-spidroin could successfully produce transparent hydrogels, suggesting the tantalizing possibility of synthetically designing custom-fitted proteins for hydrogel formation. However, all the hydrogels made in their study require at least 50 mg/mL. Fast gelation requires a high protein concentration (300 mg/mL) [16].

Building on this pioneering work, we introduced a recombinant protein engineered to feature an NT, a repeat region, and a CT. The critical feature of one of the engineered proteins is its rapid gelation capability. In conditions mimicking human body temperature, precisely at 37 °C, and at a concentration of 30 mg/mL, it can transition into a hydrogel in an astonishingly quick duration of 30 s. Upon gelation, it exhibits the formation of amyloid-like fibrils reminiscent of biological processes observed in nature. Furthermore, the multifunctional nature of this hydrogel is not just limited to its rapid formation. It showcases immense potential as a drug delivery medium, offering a controlled release mechanism. Its compatibility with the encapsulation of *E. coli* is particularly noteworthy, opening doors for potential applications ranging from probiotic delivery to targeted treatments. 

## 2. Results and Discussion

### 2.1. Fabrication of Hydrogels from Genetically Engineered Artificial Silk Proteins

**Figure 1:**
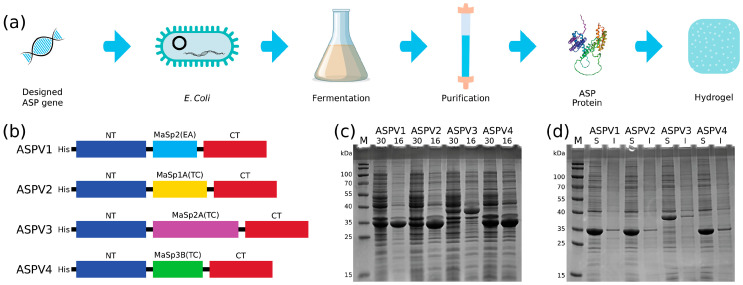
Design and recombinant production of artificial silk proteins (ASPs) for hydrogel formation. (**a**) Schematic of the recombinant ASP production process, from gene design to hydrogel formation. (**b**) Diagrammatic representation of the ASP variants engineered with different spidroin domains. All variants contain a His-tag (His), an N-terminal domain (NT) derived from *E. australis* MaSp1, a variable central repetitive region, and a C-terminal domain (CT) from *A. ventricosus* MiSps. (**c**) SDS-PAGE analysis of soluble ASP expression at 16 °C and 30 °C. (**d**) Solubility assessment of ASP variants, indicating a high degree of soluble expression. S: soluble fraction, I: insoluble fraction.

This study presents a comprehensive protocol for the generation of hydrogels utilizing artificial silk proteins (ASPs) based on the robust framework of recombinant protein technology (Figure 1a). The outlined methodology adheres to the standard recombinant protein production pipeline, initiating the conceptualization and synthesis of an ASP-encoding gene and subsequent transformation into *Escherichia coli* for ASP expression [17]. 

The synthetic gene for ASP expression is crafted following the paradigm architecture of major ampullate spidroins (MASPs) and minor ampullate spidroins (MiSps), comprising an N-terminal domain (NT) that ensures solubility, a repetitive central region, and a C-terminal domain (CT) that promotes β-sheet formation under acidic conditions [18]. Natural spidroins are highly soluble and pH-sensitive, which makes their recombinant counterparts challenging to produce. This study utilizes an innovative approach incorporating a highly soluble NT from *E. australis* MaSp1, a truncated repetitive region, and a CT from *A. ventricosus* MiSps [16]. Based on the architecture, four ASPs were designed and synthesized. ASPV1 (34 kDa) has the repeat region from *E. australis* MaSp2 (Figure 1b) [19]. The other three ASPs have the central repetitive region and are varied to include sequences from *Trichonephila clavipes* MaSp1A, MaSp2A, and MaSp3B [20], resulting in the constructs ASPV2 (34.8 kDa), ASPV3 (40.6 kDa), and ASPV4 (34.9 kDa), respectively (Figure 1b). The expression of these constructs in *E. coli* BL21(DE3) revealed an optimal protein yield at 16 °C (Figure 1c), with all variants demonstrating a high degree (more than 90%) of soluble expression (Figure 1d). Under refined flask fermentation conditions, these four ASPs were produced and purified with Ni-NTA, as shown in Supplementary Figure S1.

### 2.2. Superfast Gelation Efficiency of Engineered Artificial Silk Protein ASPV2

**Figure 2:**
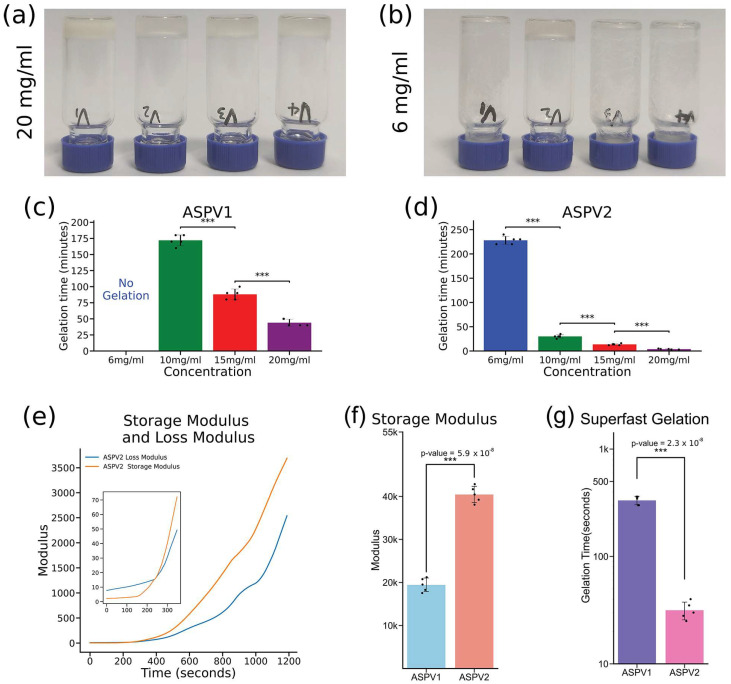
Comprehensive analysis of artificial silk proteins (ASPs) in hydrogel formation and mechanical characterization. (**a**,**b**) Visual images of hydrogel formation at 20 mg/mL (**a**) and 6 mg/mL (**b**) for ASP variants. (**c**,**d**) Gelation time analysis for ASPV1 and ASPV2 showed a concentration-dependent decrease in gelation time for both. (**e**) Storage modulus and loss modulus value during ASPV2 gelation. The inside image is a zoomed-in image to show the cross-point between storage and loss modulus. (**f**) Storage modulus difference between ASPV1 and ASPV2 after gelation. (**g**) “Superfast gelation” of ASPV2 at 30 mg/mL compared with the gelation of ASPV1 at the same concentration. Statistical significance is denoted by asterisks (*** *p* < 0.001).

The gelation kinetics of four artificial silk proteins (ASPs) were studied to pursue advanced hydrogel biomaterials. At a 20 mg/mL concentration, all four ASPs can form a hydrogel in a 240 min time scale (Figure 2a, Supplementary Figures S2 and S3). It only takes 4 min for the ASPV2 to form hydrogel and 50 min for the ASPV1 to gel. ASPV3 and ASPV4 took about 240 min to form a hydrogel. However, at a 6 mg/mL concentration, only ASPV2 can form a hydrogel in about 220 min (Figure 2b); all the other three ASPs cannot form a solidified gel in 24 h and beyond. ASPV1 exhibited a concentration-dependent gelation time, where no gelation occurred at the lowest concentration (6 mg/mL) within the experimental timeframe. As the concentration increased to 10 mg/mL, the gelation started, albeit after a protracted period, taking about 170 min to complete (Figure 2c). The gelation time decreases to 80 min at a 15 mg/mL concentration.

In contrast, ASPV2 demonstrated exceptional gelation speed, with a 20 mg/mL solution transforming into a gel within 4 min (Figure 2d), 41 min faster than ASPV1 at the same concentration. The gelation time of ASPV2 (20 mg/mL) was also confirmed by rheological data (Figure 2e), which showed the storage and loss modulus values during ASPV2 gelation. The inside image in Figure 2g showed the cross-point, an indication of gelation, at about 3.8 min, which is consistent with the gelation time measured by the inverting method. Rheological data (Figure 2f) also showed the mechanical property difference between ASPV1 hydrogel and ASPV2 hydrogel. The storage modulus, a measure of a material’s stiffness, when subjected to sinusoidal deformation of the ASPV2 hydrogel, is approximately twice the ASPV1 hydrogel. This indicates that ASPV2 has a higher mechanical strength and stiffness.

We further tested the gelation of ASPV2 at 30 mg/mL, which results in gelation in 30 s—a phenomenon we have termed “superfast gelation”. ASPV1 takes about 330 s at a 30 mg/mL concentration to solidify (Figure 2g). It is worth highlighting that previously reported silk protein requires a minimum of a 300 mg/mL concentration to achieve a 10 min gelation time [16]. Our ASPV2 only requires 1/10 of the protein concentration, resulting in 20 times faster gelation.

**Figure 3:**
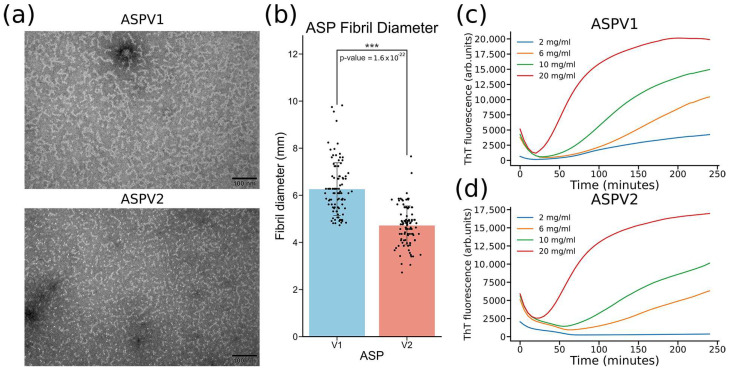
Comparative analysis of hydrogel structure formation and fibrillation kinetics between ASPV1 and ASPV2. (**a**) Transmission electron microscopy (TEM) images illustrating the nanostructured composition of hydrogels formed by ASPV1 and ASPV2. (**b**) Quantitative analysis of fibril diameters. (**c**,**d**) Thioflavin T (ThT) fluorescence kinetics curves for ASPV1 (**c**) and ASPV2 (**d**) at various concentrations, indicating fibrillation activity during hydrogel formation at 37 °C over 240 min. Statistical significance is denoted by asterisks (*** *p* < 0.001).

Transmission electron microscopy (TEM) was used to study the structure of the formed gel, which showed the formation of fibrils with or without sonication treatment on sample preparation (Figure 3a, Supplementary Figure S4). The diameter of ASPV1 nanofibrils is higher than that of ASPV2 (Figure 3b). The Thioflavin (ThT) assay was used to study the fibrillation kinetic during gelation for ASPV1 and ASPV2 (Figure 3c,d) [21]. The ThT fluorescence signal increased with the concentration when ASPV1 and ASPV2 were incubated at 37 °C. At a low protein concentration (2 mg/mL), the ThT fluorescence signal does not increase for ASPV2 (Figure 3d). The ThT fluorescence signal of ASPV1 rises to a relatively low level. At higher protein concentrations, the ThT fluorescence signal increases as time increases. After 240 min of incubation, the ThT fluorescence signal of ASPV1 at all different concentrations is higher than ASPV2. 

### 2.3. Modulation of ASPV2 Gelation Time by Ionic, pH, and Temperature, and the Enzymatic Degradation of ASPV2 Hydrogel

**Figure 4:**
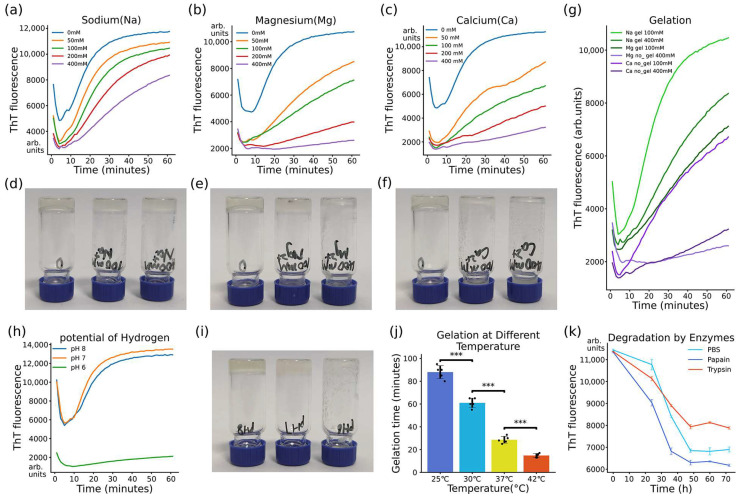
Influence of ions, pH, temperature, and enzymatic degradation on the gelation kinetics and stability of ASPV2 hydrogels. (**a**–**c**) Thioflavin T (ThT) fluorescence kinetics curves for ASPV2 fibrillation in the presence of increasing concentrations of sodium (Na^+^), magnesium (Mg^2+^), and calcium (Ca^2+^) ions. (**d**–**f**) Gelation assessment of ASPV2 with varying Na^+^, Mg^2+^, and Ca^2+^ concentrations, showing the different extents of the impact on hydrogel formation. (**g**) ThT fluorescence kinetics associated with gelation assessment at 100 mM and 400 mM for Na^+^, Mg^2+^, and Ca^2+^, correlating fluorescence intensity with gel formation. (**h**) ThT fluorescence kinetics curves for ASPV2 fibrillation under different pH conditions. (**i**) Visual depiction of hydrogel formation at pH 7 and 8 compared to inhibited gelation at pH 6. (**j**) Bar graph presenting the gelation times at various temperatures. (**k**) ThT fluorescence intensity curves showing ASPV2 hydrogel degradation in PBS, papain, and trypsin treatment over time. Statistical significance, where applicable, is indicated by asterisks (*** *p* < 0.001).

The gelation kinetics of ASPV2 were systematically studied under various ions, pH levels, and temperature conditions, followed by an assessment of hydrogel stability in the face of enzymatic degradation [22]. In the ionic studies, the ThT fluorescence assays were employed to track fibril assembly in the presence of increasing concentrations of sodium (Na^+^), magnesium (Mg^2+^), and calcium (Ca^2+^). A concentration-dependent decrease in fibrillation was apparent with all three tested ions (Figure 3a–c). As the ion concentration increases, the ThT fluorescence signal decreases. These reflect that all three ions have adverse effects on fibril assembly. Gelation formation was also tested to check the effect of ion concentration. It showed that Na^+^ (100 mM or 400 mM) has little impact on ASPV2 gelation (Figure 4d); Na^+^ (400 mM) only shows 6 min of delay of gelation compared to ASPV2 without Na^+^ (Supplementary Figure S5). The influence of sodium on the mechanical properties of ASPV2 hydrogel is pronounced, as seen in Supplementary Figure S7. While Na^+^ at 100 mM showed a negligible impact on the gelation time of ASPV2, it significantly enhances the hydrogel’s storage modulus, suggesting increased stiffness.

The hydrogel formation of the Mg^2+^ 200 mM is 11 min slower than without Mg^2+^ (Supplementary Figure S6), but Mg^2+^ 400 mM fails to form hydrogel (Figure 4e). ASPV2 cannot initiate gelation at both Ca^2+^ 100 mM and 400 mM concentrations (Figure 4f). The ThT fluorescence kinetic data (Figure 4g) corresponding to the tested gelation of three different ions at 100 mM and 400 mM showed that the ThT fluorescence signal is consistent with the hydrogel formation. 

The role of pH on gelation kinetics was evaluated, revealing that neutral to slightly alkaline conditions (pH 7.0 and 8.0) are preferred conditions for gel formation, but there is no gelation time difference between ASPV2 pH7.0 and ASPV2 pH8.0 (Supplementary Figure S8). Notably, at pH 8.0, the hydrogel is more transparent than pH 7.0. In contrast, an acidic environment (pH 6) significantly impeded the process (Figure 4h,i). Investigating the influence of temperature on gelation revealed that 37 °C, a temperature consistent with human body conditions, favored rapid gel formation. A lower (25 °C) temperature resulted in slower gelation processes (Figure 4j), while a higher (42 °C) temperature resulted in the fastest gelation. 

The structural robustness of ASPV2 hydrogels was challenged with proteolytic enzymes [23]. The hydrogels demonstrated degradation in phosphate-buffered saline (PBS), whereas treatment with papain led to a notably faster reduction in the ThT fluorescence signal, indicating enzymatic hydrogel breakdown over time (Figure 4k). However, trypsin showed no enhanced degrading effect on ASPV2 hydrogel.

### 2.4. ASPV2 Hydrogel as a Platform for Protein Release and Bacterial 3D Culture

**Figure 5:**
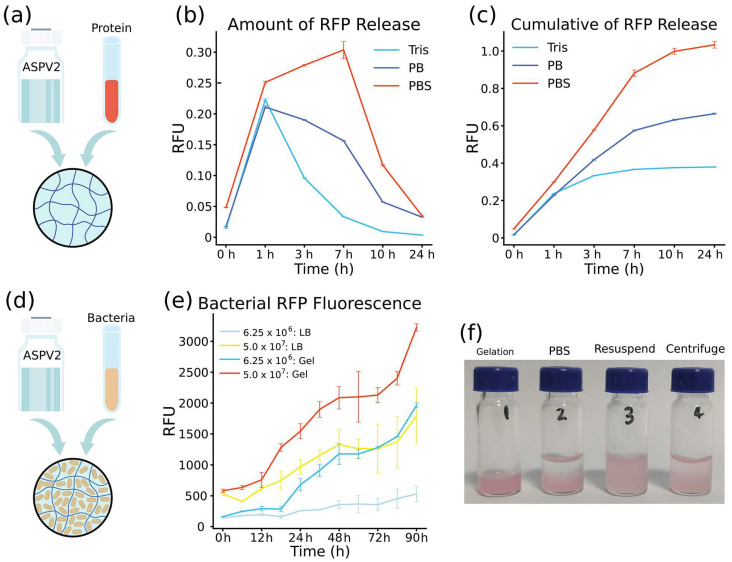
Controlled red fluorescent protein (RFP) release from ASPV2 hydrogel and its application as a cultivation 3D matrix for *E. coli*. (**a**) Illustration of the encapsulation process of RFP within the ASPV2 hydrogel matrix by mixing equal volumes of ASPV2 (20 mg/mL) and RFP. (**b**) Graph depicting the immediate release kinetics of RFP in Tris, PB, and PBS buffer. (**c**) The accumulated release profile of RFP shows buffer composition’s impact on release behavior. (**d**) Schematic of the encapsulation of *E. coli* in the ASPV2 3D hydrogel matrix with subsequent RFP expression from an IPTG inducible plasmid. (**e**) Comparing RFP expression levels over 90 h in *E. coli* cultivated in an LB medium and within the ASPV2 3D hydrogel. (**f**) Sequential photographs illustrating the processing steps involved in cell recovery from hydrogel. The images show the hydrogel initially post-gelation (1), after PBS buffer wash (2), following resuspension (3), and after a centrifugation step (4).

The potential of ASPV2 hydrogel as a controlled release matrix was evaluated using red fluorescent protein (RFP) as a model therapeutic agent [24,25]. To make the hydrogel carrying RFP, mix the same volume of ASPV2 protein (20 mg/mL) and RFP (5 mg/mL), and then incubate at 37 °C for 30 min (Figure 1a). The release kinetics indicated an initial burst release in Tris or PB, with a significant amount of RFP released within the first hour. Subsequent release rates decreased, suggesting a transition to a more controlled release phase (Figure 5b). The release pattern differs in PBS, which has a high release level from 1 to 7 h, and then transits to slow release. The cumulative protein release in PBS reaches 100%, while it only achieves 35% or 60% percent of release for Tris and PB, respectively. Cumulative release profiles demonstrated that the medium’s composition affects release behavior, with the release in PBS showing a consistent increase, while Tris or PB buffer enters the plateau stage as early as 3 h (Figure 5c).

The ASPV2 hydrogel was further tested as a 3D matrix for *E. coli* expressing RFP from an IPTG inducible plasmid [26,27,28]. The bacteria were successfully encapsulated and maintained within the hydrogel matrix by incubating at 37 °C for 30 min after mixing an equal volume of ASPV2 protein (20 mg/mL) and bacterial culture. Induced RFP expression was observed over 90 h. This steady increase in fluorescent signal suggests the hydrogel supports bacterial viability and is conducive to the expression system (Figure 5e). Moreover, a higher RFP signal was observed in hydrogel than LB in two different cell concentrations. 

The possibility of recovering cells from the 3D matrix hydrogel was also tested [29]. A double volume (400 μL) of PBS was added to the gel (200 μL), and then the gel was pipetted up and down until there was no obvious gel pellet (Figure 5f). The color intensity changes in the hydrogel post-PBS treatment and the subsequent resuspension step indicated cell release. This was further confirmed by the clear upper layer supernatant following centrifugation, underscoring the hydrogel’s ability to release cells by pipetting (Figure 5f).

### 2.5. Discussion

This study presents an illustrative case of how synthetic biology can be utilized to engineer materials with specific desired characteristics. The ASP was designed to mimic the structure of natural spider silk proteins, known for their exceptional mechanical properties, including tensile strength and elasticity. By designing or selecting (from spider silk proteins) amino acid sequences within the central repetitive domain of the proteins, we can modulate the physical characteristics of the hydrogels formed from the designed protein. This level of control over the material properties opens up many biomedical applications, ranging from drug-releasing tools to creating a 3D culture for biomedical research and therapeutic applications. 

This study successfully produced four ASP variants based on the NT–repeats–CT architecture. By optimizing growth conditions and purification processes, we ensured that the ASP could be made with high purity and sufficient soluble ASP for practical applications. These four ASPs showed different gelation times, mainly due to the protein sequence of the repeat region. The repeat region of ASPV1 is from *E. australis* MaSp2, which differs from the reported His-NT2RepCT with a repeat region from *E. australis* MaSp1 [16]. The repeat regions of ASPV2, ASPV3, and ASPV4 are from *Trichonephila clavipes* MaSp1A, MaSp2A, and MaSp3B, respectively. Alphafold2 structure modeling of the four ASPs showed structure difference in the middle region (Supplementary Figure S9) [30], but it is hard to relate the predicted structure to the gelation time difference. These four ASPs showed different gelation times at both 6 mg/mL and 20 mg/mL concentrations. Further study of ASPV1 and ASPV2 gelation showed that both ASPs showed concentration-dependent hydrogel formation. The gelation time of ASPV2 (20 mg/mL) is almost the same for both measures by inverting method and using the cross-point between storage and loss modulus. The rheological data also showed that ASPV2 hydrogel has higher stiffness than ASPV1 hydrogel. To our surprise, ASPV2 can form a hydrogel in 30 s at 37 °C and a 30 mg/mL concentration, termed “superfast gelation”. This body temperature rapid gelation character has potential clinical applications [31,32], such as delivering hemostatic agents in trauma care or sealants in surgical procedures. Compared to previously reported fast gelation recipes, ASPV2 takes advantage of self-assembly, which does not require additives.

The nanofibrillar structure of the hydrogels, revealed by TEM analysis, provides insights into the molecular arrangement that underpins the material’s mechanical properties. The diameter of fibrils in hydrogels, influenced by protein sequence adjustments, directly affects the gel’s texture and porosity. Smaller fibril diameters lead to a smoother texture. Porosity, crucial for functions like liquid absorption and nutrient permeability, is also determined by fibril size, with larger fibrils creating more significant gaps and, thus, higher porosity. This nanoarchitecture is reminiscent of the hierarchical organization of natural extracellular matrix (ECM) components, suggesting that ASP hydrogels could serve as ECM mimics for 3D cell culturing or tissue engineering purposes [33]. The variation in fibril diameters among the ASP variants indicates that adjusting the protein sequence can tailor the hydrogel’s texture and porosity. Ionic concentrations, pH, and temperature affect ASPV2 hydrogel formation. We can design hydrogels with different properties and speeds by understanding how these factors affect gelation kinetics and stability. Metal ions were known to modulate the stiffness and elastic properties of formed hydrogel [34,35]. This study showed that ASPV2 hydrogel formation can tolerate high Na^+^ concentrations. With a 100 mM Na concentration, the gelation time did not increase, but the storage modulus increased by about 1.6 times, indicating an increase in hydrogel stiffness. Low Mg^2+^ concentration mildly affects gelation, but Ca^2+^ strongly negatively affects ASPV2 gelation. Na^+^ and Mg^2+^ would be good candidates for adjusting the hydrogel strength without delaying gelation. The influence of temperature on gelation kinetics also has practical implications for storing and handling these materials [36]. The rapid gelation at physiological temperatures, paired with the more gradual transition at lower temperatures, provides flexibility in the administration of the hydrogel. This feature could allow the hydrogel to be injected as a liquid at room temperature and then transition to a solid phase once it reaches the target site within the body, ensuring minimal invasiveness and enhanced patient comfort. The enzymatic degradability of ASPV2 hydrogels is a critical feature for their potential use as biodegradable implants [37]. The ThT fluorescence data showed that PBS itself can help hydrogel degradation. The enzyme papain can speed up the degradation process, while trypsin has a similar effect as PBS. This indicates that ASPV2 degradation is enzyme-specific, which helps modulate the hydrogel in actual application. This tunable biodegradability could be beneficial in drug delivery applications, where the hydrogel could provide a sustained release of therapeutics over a period that aligns with the treatment regimen, followed by biodegradation to avoid the need for the removal process. In summary, the gelation of ASPV2 requires specific, mild conditions to ensure optimal formation. Firstly, the process occurs at 37 °C, corresponding to the average human body temperature, which is ideal for the proper folding and interaction of protein chains necessary for gel formation. This temperature is often chosen in biological experiments, as it aligns with the optimal conditions for many biochemical processes in mammals. Secondly, a pH level of 7.0, which is neutral, is maintained. This neutrality is crucial, as it prevents the denaturation or unwanted aggregation of proteins, ensuring that the amino acids within the protein maintain their correct ionization state and structural integrity. Lastly, a Tris HCl buffer stabilizes the environment, maintaining a constant pH during gelation. Tris HCl is a standard biochemical buffer that helps preserve the ionic strength and osmotic balance, which is essential for facilitating the correct molecular interactions during the hydrogel formation. These conditions collectively create an ideal environment for ASPV2 to undergo effective and stable gelation. 

The hydrogel’s ability to release red fluorescent protein (RFP) in a controlled manner is of significant interest for therapeutic applications. The observed initial burst release (from Tris or PB) could be advantageous for delivering a rapid therapeutic effect, followed by a more sustained release profile ensuring prolonged protein delivery. The varying kinetics in Tris, PB, and PBS highlight the hydrogel’s responsiveness to different ionic strengths and buffer compositions, potentially allowing for the tailoring of release profiles based on therapeutic needs. Moreover, the 100% cumulative release in PBS suggests that the hydrogel can be fully utilized without leaving residual materials that could lead to complications or reduced efficacy [38]. While variations in release kinetics were noted in Tris, PB, and PBS, it is crucial to recognize that the release environment in vivo is not adjustable. Therefore, future work can focus on designing the hydrogel material to tailor release profiles, ensuring they are adaptable to the human body’s fixed ionic conditions and physiological environment to meet therapeutic needs effectively.

For bacterial 3D cultivation, hydrogel demonstrates excellent potential in maintaining bacterial viability and promoting protein expression. The steady increase in the fluorescent signal over 90 h implies that the hydrogel matrix does not hinder bacterial growth or the IPTG-induced expression of RFP. Moreover, the RFP signal is higher than in LB, indicating that bacterial growth was promoted or RFP expression was enhanced. This is particularly notable as it suggests that hydrogel can provide a suitable environment for sustained bacterial activity and production, which is crucial for research and industrial applications. However, it is important to acknowledge that the results based on *E. coli* may not directly translate to more complex cell types, such as neural progenitor cells. Future studies involving a broader range of cell types, including mammalian cells, would be necessary to fully understand the hydrogel’s potential in more diverse and clinically relevant applications. Moreover, the feasibility of cell recovery from the hydrogel is also demonstrated, which is essential for downstream processing and applications where cell reuse or product recovery is required. The clear supernatant following centrifugation confirms that cells can be released efficiently, which is critical for practical applications. 

Despite its promising features, the study recognizes several limitations. While beneficial for specific applications, the ASPV2 hydrogel’s susceptibility to enzymatic degradation may limit its use in long-term implantable devices [39]. Additionally, the current study’s scale and scope may not fully capture the complex interactions in vivo, where immune response, tissue integration, and long-term stability play significant roles [40].

Future investigations could focus on tuning the mechanical strength to fit biomedical applications and enhancing the stability of the hydrogel to reduce enzymatic degradation rates without compromising its biocompatibility and biodegradability. Developing ASPV2-based organoid production will be particularly interesting in future high-throughput drug screens. Long-term in vivo studies are essential to understand the hydrogel’s performance in a living organism and assess its potential immunogenicity. Furthermore, scaling up the production and examining the storage stability of the hydrogel are critical steps toward its commercialization and clinical translation. Exploring the hydrogel’s capabilities in co-delivering multiple therapeutic agents, its application in wound healing and tissue engineering, and its use as a scaffold for cell growth could widen its scope of application.

## 3. Conclusions

In conclusion, ASPV2 hydrogel represents a significant advancement in biomaterials. Our study demonstrates the promising potential of hydrogel for minimally invasive therapeutic applications, particularly due to its rapid gelation at physiological temperatures. While the hydrogel’s enzymatic degradability poses challenges for long-term implantation, it benefits applications requiring short-term intervention and natural biodegradation. The hydrogel’s capacity to maintain bacterial viability and enable protein expression opens new avenues in biotechnology and pharmaceutical research. 

However, we recognize that our investigation into how ionic, pH, and temperature adjustments affect gelation time is just the beginning. Further research is essential to comprehensively assess and fine-tune other critical properties, such as material stiffness, to fully harness the hydrogel’s capabilities for a broader range of biomedical applications. Future work will also focus on optimizing hydrogel stability for organoid cultivation and high-throughput drug screening, with in vivo studies being crucial for assessing its clinical viability. The transition from the bench to bedside for ASPV2 hydrogels will depend on successful scale-up, storage stability, and safety evaluations. This highlights its promise as a next-generation tool for biomedical applications.

## 4. Materials and Methods

### 4.1. Bacteria and Growth Media

*E. coli* host strains DH5α and BL21(DE3) were procured from NEB (Ipswich, MA, USA). *E. coli* cultures were grown in Luria–Bertani (LB) broth or an LB agar from Sangon Biotech (Shanghai, China). Bacteria containing the ASP-expressing plasmids were cultured and supplemented with 100 µg/mL of ampicillin. Bacteria with pWF06 plasmid were cultured and supplemented with 50 μg/mL Kanamycin.

### 4.2. Plasmid Construction

The pASPV1, pASPV2, pASPV3, and pASPV4 plasmids used pETDuet-1 as the backbone, and the insert part was synthesized by Gentlegen, Suzhou, China. Plasmid information was provided in the supplementary seq 1 to 4. The synthesized plasmid was propagated in *E. coli* DH5alpha and transferred into *E. coli* BL21(DE3) for protein expression.

Gibson assembly was used to clone the pWF06 plasmid, which expresses red fluorescent protein (RFP). The backbone was amplified with PCR using primer LC98 (5′-TTAACCTAGGCTGCTGCCAC-3′) and primer LC368 (5′-CATGGTATATCTCCTTATTAAAGTTAAAC-3′) from pRSFDuet-1 plasmid [41]. The insert was amplified from template pYC1000-eforRED using primer CYJ83 (5′-GTTTAACTTTAATAAGGAGATATACCATGTCAGTGATTAAGCAGGTAATGAAGA-3′) and primer LC771 (5′-GTGGCAGCAGCCTAGGTTAATTATGGGAGAGCCTTCGGCA-3′) [42]. 

### 4.3. Protein Expression and Purification

*E. coli* BL21(DE3) glycerol stocks with the corresponding plasmids were taken from −80 °C, inoculated into an LB medium (3 mL), and cultured overnight at 37 °C and 180 rpm. The following day, the overnight culture was inoculated at a 1:100 ratio into a 1 L LB medium. When OD600 reached 0.5–0.6, the culture was transferred to 16 °C and 180 rpm for 20 min before adding IPTG (a final concentration of 0.5 mM). The culture was kept at 16 °C and 180 rpm until harvest. To harvest the culture, centrifuge at 4 °C, 3200× *g*, for 12 min to obtain cell pellets. 

For protein purification, the cell pellet was resuspended in lysis buffer (50 mM Tris-HCl, pH 8.0, 100 mM NaCl, 5 mM 2-Mercaptoethanol, 0.1% Tween-20, 1 mM imidazole) using a ratio of 1 g wet bacteria to 5 mL buffer. Following complete resuspension, the cells were then sonicated (100 w for 1 min, 2 s ON, 2 s OFF). The lysate was centrifuged at 4 °C and 12,000× *g* for 20 min. The supernatant was filtered through a 0.22 μm aqueous syringe filter (Millipore ExpressPEG Membrane, Burlington, MA, USA). The filtrate was then loaded onto a Ni-NTA column, and the target protein was eluted with 250 mM imidazole. All purification procedures were performed using an AKTA Explorer 100 system from GE Healthcare (Chicago, IL, USA). The purified protein was checked with SDS-PAGE gel and Coomassie Staining and quantified with a Bradford assay.

The eluted protein was dialyzed in 50 mM Tris-HCl, pH 8.0, using an MWCO 14 kDa dialysis membrane for 24 h, with the dialysis solution changed every 12 h. The dialyzed protein was concentrated using an ultra-filtration column (15 mL, 10 kDa MWCO, Sartorius, Göttingen, Germany) with approximately 45 min of centrifuge. The final protein concentration was quantified with a Bradford assay. The high-concentration protein formed a transparent, slightly yellow, viscous solution. The high-concentration protein solution was stored at −80 °C.

### 4.4. Sodium Dodecyl-Sulfate Polyacrylamide Gel Electrophoresis (SDS-PAGE) and Coomassie Staining

Samples were pelleted with 4000× *g* 10 min of centrifuge and then resuspended in a mixture of 2× Laemmli Sample Buffer (Bio-Rad, Hercules, CA, USA), β-mercaptoethanol, and sample at a 10:1:9 ratio. Samples were incubated for 5 min at 95 °C before loading into 12% (*w*/*v*) Tris-Glycine SDS precast polyacrylamide gels (Vazyme, Nanjing, China). The gel electrophoresis was run in a Mini Gel Electrophoresis System (Bio-Rad) with a constant voltage set at 80 V for the stacking gel and 120 V for the separating gel. Following electrophoresis, a standard Coomassie stain procedure was used to stain the gel, which was imaged using the Gel Doc system (Bio-Rad, Hercules, CA, USA). 

### 4.5. Protein Gelation

After taking the protein solution out of the −80 °C freezer and allowing it to thaw on ice (Do not thaw at room temperature), 200 μL of the protein solution was slowly transferred into a 1.5 mL transparent liquid chromatography sample vial using a pipette. The lid was closed to prevent evaporation. The samples were immediately placed in a 37 °C incubator for incubation, and the protein gelation was observed by inverting the vials, recording the gelation time, and taking photos. Samples that did not gel were incubated at 37 °C for at least 6 h.

### 4.6. TEM (Transmission Electron Microscopy)

A total of 20 mg/mL of SPV1 and SPV2 solutions were incubated at 37 °C for 2 h until complete gelation. The hydrogel was then freeze-dried for 24 h (at −60 °C, 100 mTorr), and then a dilution buffer (50 mM Tris-HCl) was used to resuspend the freeze-dried hydrogel thoroughly by vortex (for 5 min) and sonication (100 W, 2 min, 10 s ON, 5 s OFF). The resuspended hydrogel was further diluted 10 folds with dilution buffer, and 5 μL of the diluted sample was dropped onto a copper grid. After waiting a few minutes, the excess sample was removed from the edge of the droplet with blotting filter paper. The sample was washed twice with 5 μL MilliQ water and then stained with 1% phosphotungstic acid (PTA) for 2 min. Excess stain was removed, and the grid was air-dried before observation under an electron microscope (JEM-1400Flash, JEOL, Tokyo, Japan). The settings were as follows: lattice resolution (0.20 nm), point resolution (0.38 nm), acceleration voltage (40 Kv~120 kV), and acceleration voltage stability (2 ppm/min). The magnification ranged from 100 to 40,000 times. The diameters of ASPV1 and ASPV2 fibers were measured using ImageJ 1.54d software (n = 100).

### 4.7. ThT Assay

Add ThT (stock concentration 1 mM) to the pre-prepared ASPV1 or ASPV2 protein (10 mg/mL) to a final concentration of 25 μM. Add 100 μL of the protein–ThT mixture to a Corning 96-well black plate, and then seal the plate with a transparent film to avoid evaporation. Measure the change in ThT fluorescence during the gelation process under static conditions at 37 °C. Measure the fluorescence signal with 436 nm excitation and 482 nm emission setting.

### 4.8. Bradford Assay 

Protein concentrations were quantified using the Detergent Compatible Bradford Protein Assay Kit (Vazyme, Nanjing, China). The Bradford assay starts by setting up a standard curve using bovine serum albumin (BSA), ranging from 0.025 to 0.125 mg/mL. Protein samples with different amounts of NaCl were mixed with the 1 mL Bradford reagent for quantification, and then 200 μL of each mixture was transferred into a transparent 96-well plate. Absorbance was measured at 595 nm using a Tecan Infinite^®^ 200 PRO Microplate Reader. The protein concentration of the unknown samples was calculated by comparing their absorbance values to those of the BSA standard curve.

### 4.9. Hydrogel Degradation Experiment

Dilute the pre-prepared protein solution to 10 mg/mL and add ThT to a final concentration of 25 μM. Mix well and transfer 100 μL to a Corning 96-well black plate with the 96-well sealed with transparent film. Incubate at 37 °C in an incubator for 2 h until fully gelled. Then, individually add 100 μL of 1× PBS, 0.1 mg/mL papain, or 0.1 mg/mL trypsinwith the top sealed. Then, incubate it at 37 °C for 24 h. The ThT fluorescence signal was measured every 12 h until 72 h. The change in the ThT fluorescence signal was used to monitor the degradation capability of different reagents on the formed hydrogel.

### 4.10. Hydrogel Protein Releasing Test

A total of 20 mg/mL ASPV2 protein mixes with red fluorescent protein (RFP from Novus Biologicals, Littleton, CO, USA) in a 1:1 volume ratio and transfers 400 μL of the mixture to each well of a 24-well plate. Incubate at 37 °C for 30 min. Then, add 1 mL of different buffer solutions (10 mM Tris-HCl pH 7.4, 10 mM PB pH 7.4, 10 mM PBS pH 7.4) to each well and test the RFP fluorescence release at intervals of 0, 1, 2, 4, 7, 10, and 24 h. Sample 100 μL of the supernatant into a transparent 96-well plate for RFP fluorescence measurement (excitation: 521 nm, and emission: 592 nm), discard the excess supernatant, and then add 1 mL of the fresh buffer after each measurement time point. 

### 4.11. Hydrogel-Based 3D Bacterial Culture and Recovery

Take the *E. coli* BL21(DE3) strain expressing RFP (pWF06 plasmid) from −80 °C and inoculate it into a 3 mL LB medium containing Kanamycin (50 mg/L). Culture it overnight at 37 °C and 180 rpm. The following day, inoculate the overnight culture into 20 mL LB medium at a 1:100 ratio and continue to culture at 37 °C and 180 rpm. When the OD600 reaches about 0.5, add the inducer IPTG (final concentration 0.5 mM) and then incubate at 30 °C and 180 rpm until the medium appears slightly red to the naked eye (at about OD600 5.0). A total of 20 mg/mL ASPV2 protein (with 0.5 mM IPTG) mixes with the above *E. coli* (with different amounts of dilution to achieve a final 6.25 × 10^6^ cells and 5 × 10^7^ cells, respectively) in a 1:1 volume ratio. Then, add 100 µL of the protein–bacteria mixture to the wells of a 96-well plate and incubate at 37 °C for 1 h for gelation. Then, add 50 uL LB medium to cover the formed hydrogel to avoid evaporation and seal the plate with transparent film. The control groups had the same amounts of cells with LB culture. RFP signal (excitation: 521 nm, and emission: 592 nm) was measured along a 90 h time course.

To set up the 3D culture in a chromatography vial, mix 200 µL of an RFP-expressing overnight culture with an equal volume of ASPV2 protein for gelation and recovery in a liquid chromatography vial, and then add it to a 1 mL vial. Incubate at 37 °C for 1 h. Once fully gelled, add 400 µL of 1× PBS, resuspend by pipetting, and centrifuge. Photos were taken during this process.

### 4.12. Rheological Measurements

The dynamic rheological properties of ASPV1 and ASPV2 were measured by an MCR301 rheometer. All tests used parallel plates had a diameter of 25 mm and a gap of 0.049 mm. The system temperature was set to 37 °C. Use a syringe to carefully add the protein solution (100 uL) to the center of the test bench. Ultrapure water was placed on the outer edge of the plate to prevent the water from evaporating. Frequency scanning was performed at 0.001–100 Hz and a step of 1%. All measurements were performed for 20 min.

### 4.13. Alphafold2 Structure Modeling

A Google colab notebook (link below) was used for AlphaFold2 and MMseqs2-based structure modeling for the 4 ASPs. The default setting was used for structure modeling (https://colab.research.google.com/github/sokrypton/ColabFold/blob/main/AlphaFold2.ipynb). We accessed the website on 7th February 2023.

### 4.14. Data Analysis

Data were obtained from at least three independent experiments, and all variables were expressed as mean ± standard deviation (SD). Differences between experimental groups were analyzed using Student’s *t*-test. Single, double, and triple asterisks represent *p* < 0.05, 0.01, 0.001, respectively, and *p* < 0.05 was considered statistically significant. All statistical analyses were performed using SPSS 12.0 software (SPSS Inc., Chicago, IL, USA).

## Data Availability

All datasets generated for this study are included in the article/Supplementary Material. Further inquiries can be directed to the corresponding author.

## Supplementary Materials

The following supporting information can be downloaded at https://www.mdpi.com/article/10.3390/gels10010069/s1, Figure S1: Analysis of protein purification stages for ASPV1, ASPV2, ASPV3, and ASPV4; Figure S2: Gelation Time of ASP Proteins at 20 mg/mL Concentration; Figure S3: Visual Representation of Gelation Process for ASPs at Different Time Intervals; Figure S4: Transmission electron microscopy (TEM) images displaying the nanofibrillar structures of hydrogels formed by ASPV1 and ASPV2 (Samples were prepared without sonication); Figure S5: Impact of Sodium Concentration on Gelation Time; Figure S6: Influence of Magnesium Concentration on Gelation Time; Figure S7: Effect of Sodium on the Storage Modulus of ASPV2; Figure S8: Gelation Time of Protein Solution at Different pH Levels; Figure S9: Predicted 3D Structures of ASPs by AlphaFold2.
