# Supplementary material for: Superfast Gelation of Spider Silk-Based Artificial Silk Protein
Source: Gels. 2024 Jan 17;10(1):69. doi: 10.3390/gels10010069 (PMC10815891; doi:10.3390/gels10010069)
Supplement: Supplementary file 1 [file gels-10-00069-s001.zip › gels-2756991-supplementary.pdf]

Superfast Gelation of Spider Silk-Based Artificial Silk Protein

Fan Wen[1], Yu Wang[1], Bowen Tu[2], Lun Cui[1, 3,*]

1 CCZU-JITRI joint Bio-X Lab, School of Pharmacy & School of Biological and Food Engineering, Changzhou University, 213164, Changzhou, Jiangsu Province, China

2 Pathogenic Biological Laboratory, Changzhou Disease Control and Prevention Centre, Changzhou Medical Centre, Nanjing Medical University, 213000, Changzhou, Jiangsu Province, China

3 Biomaterials Lab, Changzhou AiRiBio Healthcare CO., LTD, 213164, Changzhou, Jiangsu Province, China

*Corresponding Author(s), E-mail(s): luncui@cczu.edu.cn

Contributing authors, E-mail: Fan Wen: wenfan25@outlook.com

Yu Wang: wyu30086@gmail.com

Bowen Tu: tbwchangzhou@163.com

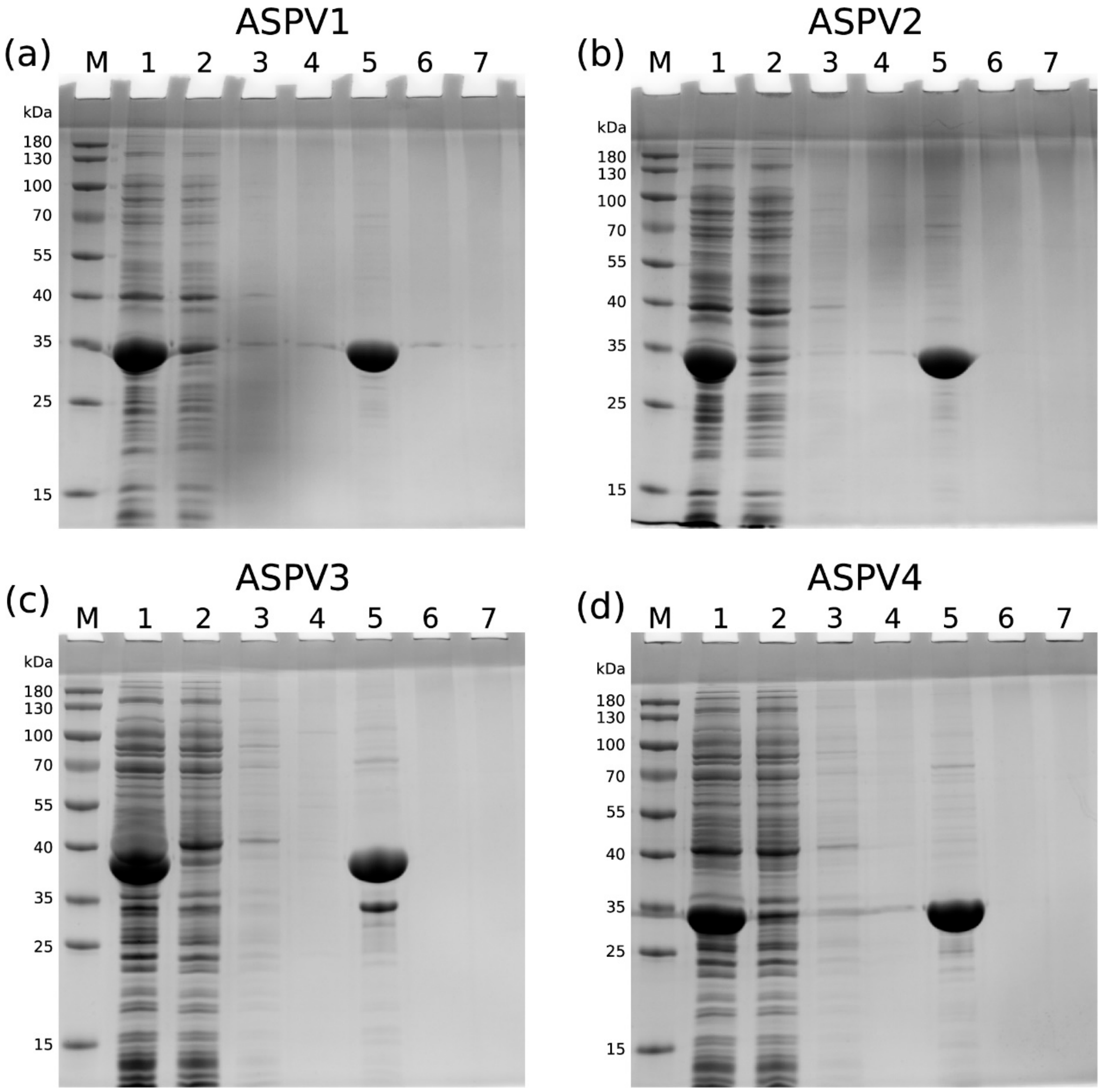

Figure S1. Analysis of protein purification stages for ASPV1, ASPV2, ASPV3, and ASPV4. SDS-PAGE was performed to assess the purification process of four different proteins designated as ASPV1 (a), ASPV2 (b), ASPV3 (c), and ASPV4 (d). Each panel represents a distinct protein purification profile. Lane M contains molecular weight markers with sizes indicated in kilodaltons (kDa). Lane 1 shows the initial cell lysate. Lane 2 contains the flow-through fraction from the binding buffer. Lanes 3 display the flow-through from the wash buffer. Lanes 4 to 7 represent successive elution fractions (Elute 1-4) with each elute volume being 5 ml, illustrating the proteins eluted during the purification process.

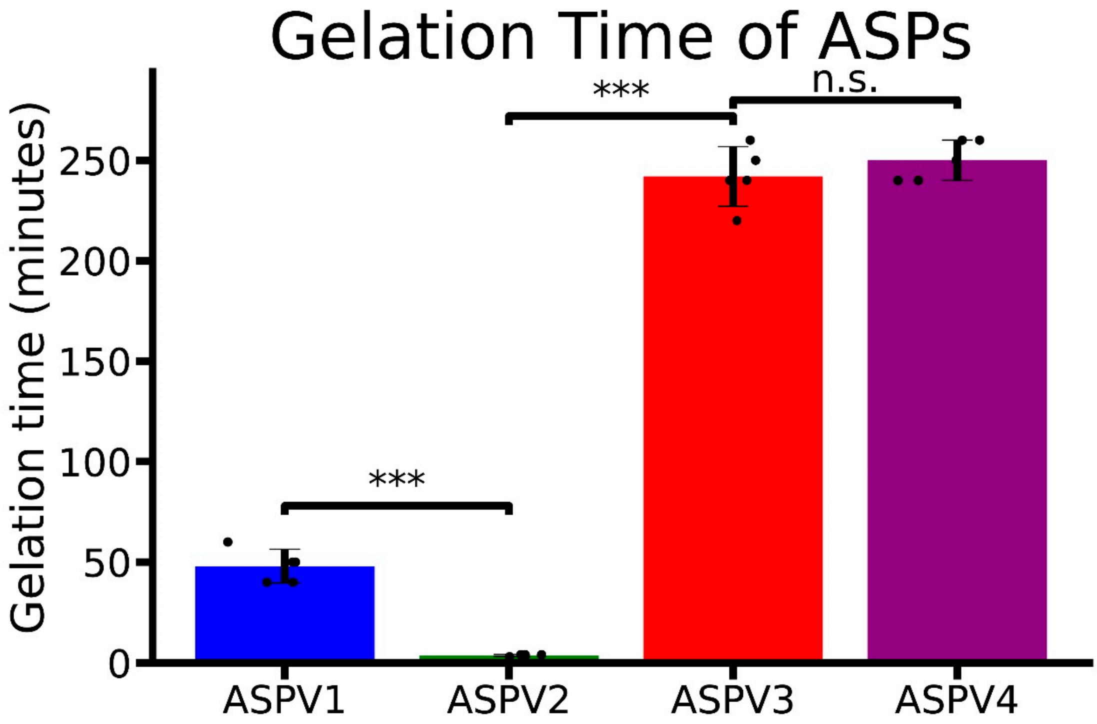

Figure S2. Gelation Time of ASP Proteins at 20 mg/ml Concentration. ASPV1 shows a significantly longer gelation time compared to ASPV2, indicated by the triple asterisks, denoting a p-value < 0.001. ASPV3 and ASPV4 have markedly increased gelation time compared to ASPV2, also indicated by triple asterisks. No significant difference (n.s.) in gelation time is observed between ASPV3 and ASPV4. Error bars represent standard deviation.

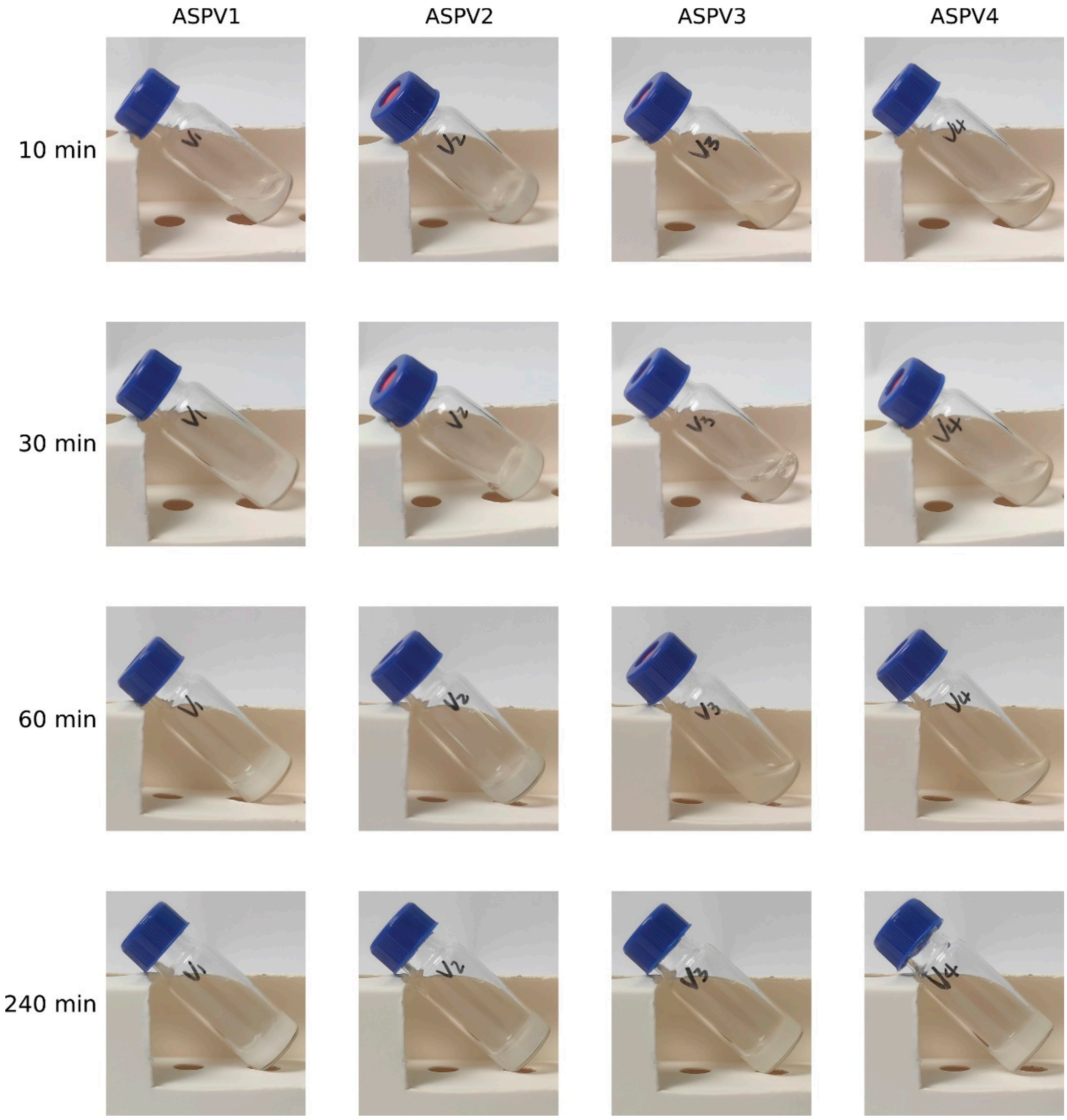

Figure S3. Visual Representation of Gelation Process for ASPs at Different Time Intervals. The series of images demonstrates the gelation of ASPV1, ASPV2, ASPV3, and ASPV4 proteins over time at a concentration of 20 mg/ml. Each row represents time points at 10, 30, 60, and 240 minutes post-initiation of the gelation process. The columns correspond to the different ASP proteins. At 10 minutes, solid hydrogel already formed for ASPV2. At 60 minutes, ASPV1 exhibits full gelation. At 240 minutes, complete gelation is observed for all ASPs.

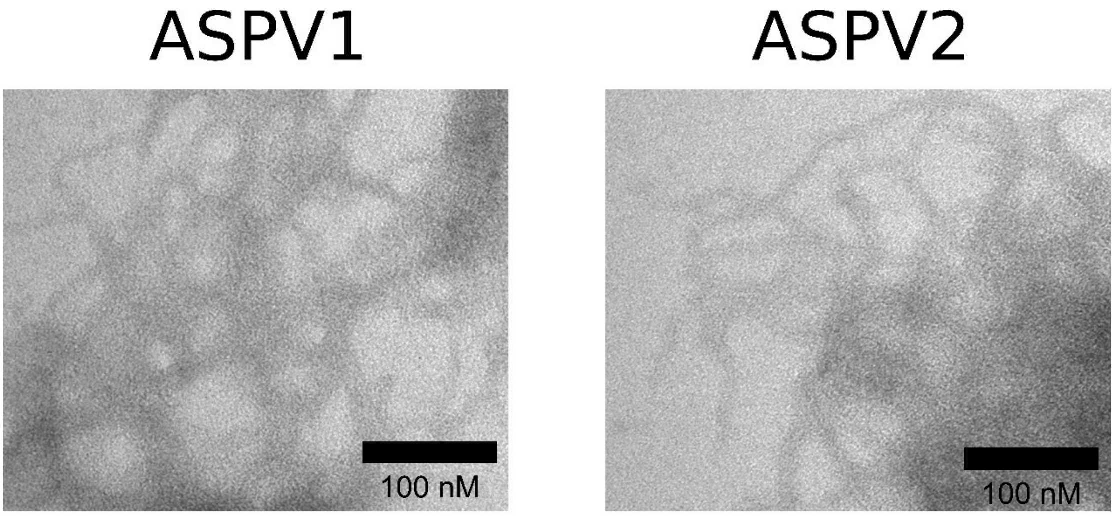

Figure S4. Transmission electron microscopy (TEM) images displaying the nanofibrillar structures of hydrogels formed by ASPV1 and ASPV2 (Samples were prepared without sonication).

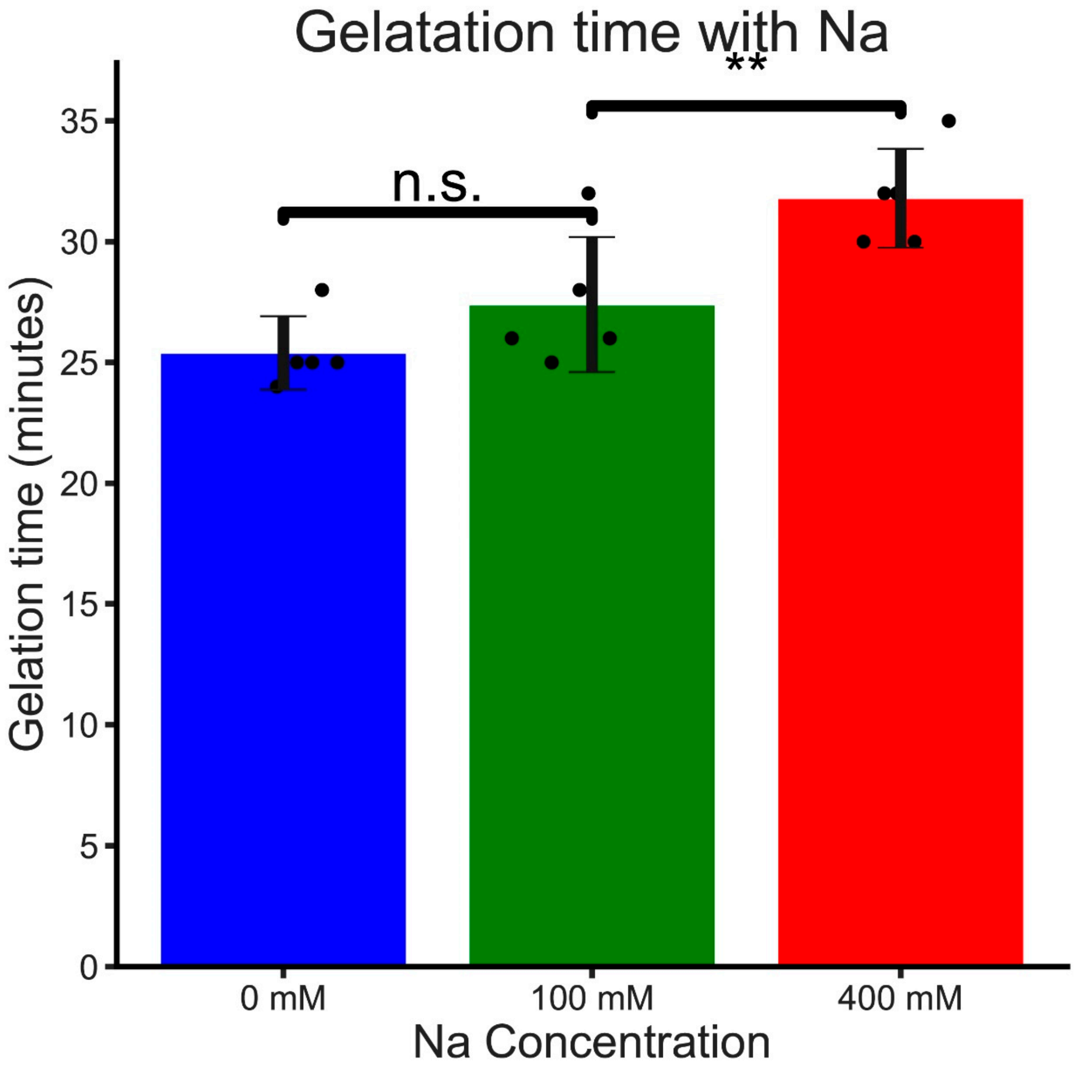

Figure S5. Impact of Sodium Concentration on Gelation Time. The bar for 0 mM Na shows the shortest gelation time. A non-significant difference (n.s.) is observed between 0 mM and 100 mM Na concentrations. However, a significant increase in gelation time is noted at 400 mM Na concentration, as indicated by double asterisks (**), corresponding to a p-value < 0.05. Error bars indicate standard deviation.

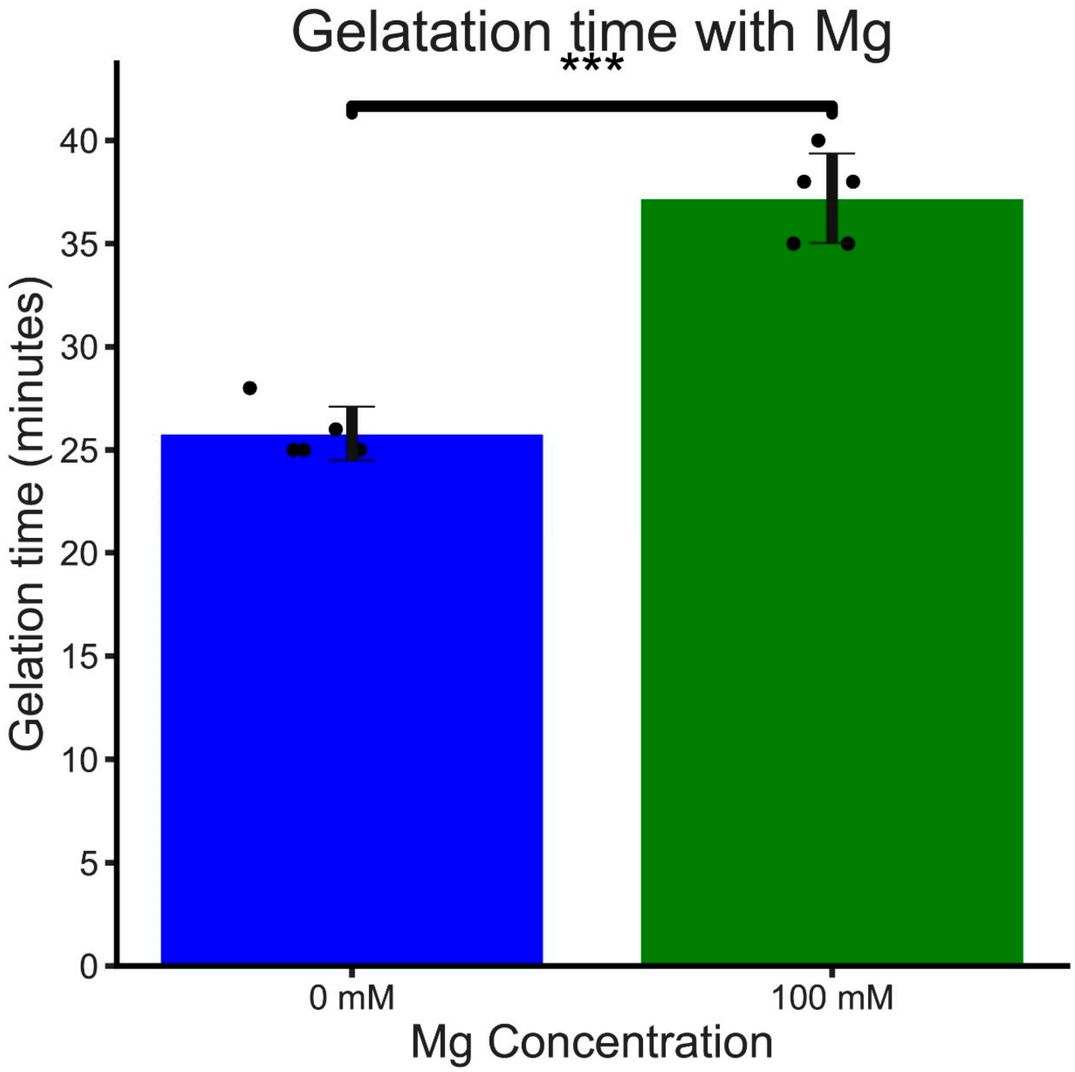

Figure S6. Influence of Magnesium Concentration on Gelation Time. The presence of 100 mM Mg significantly increases the gelation time compared to the absence of Mg, as shown by the green bar and denoted by the triple asterisks (***), indicating a p-value < 0.001. Error bars represent the standard deviation.

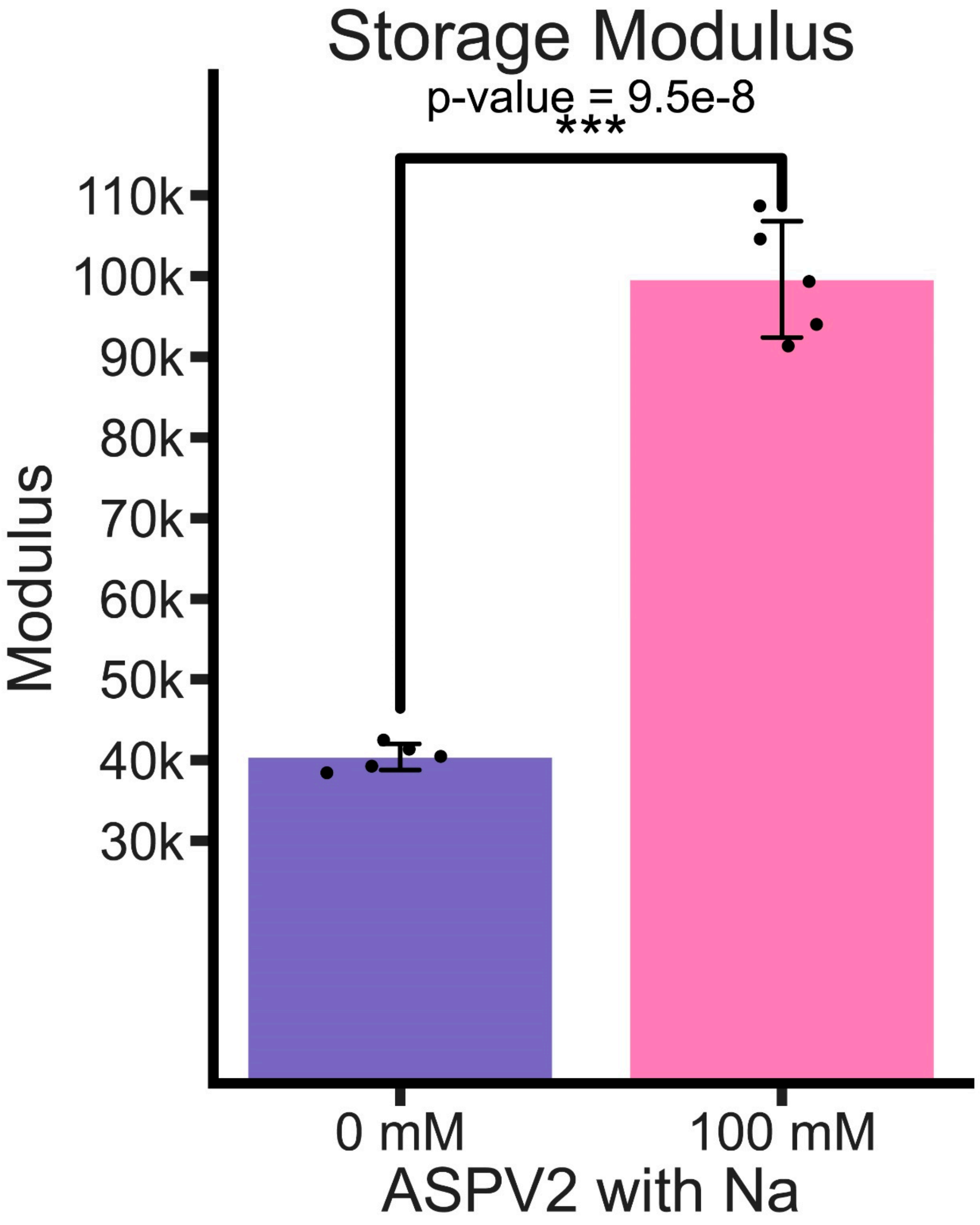

Figure S7. Effect of Sodium on the Storage Modulus of ASPV2. The left bar (purple) shows the storage modulus of ASPV2 without sodium (0 mM Na), and the right bar (pink) indicates the storage modulus with 100 mM sodium. Error bars depict the standard deviation, the statistical significance between the two conditions is denoted by asterisks (***), with a p-value of 9.5e-8.

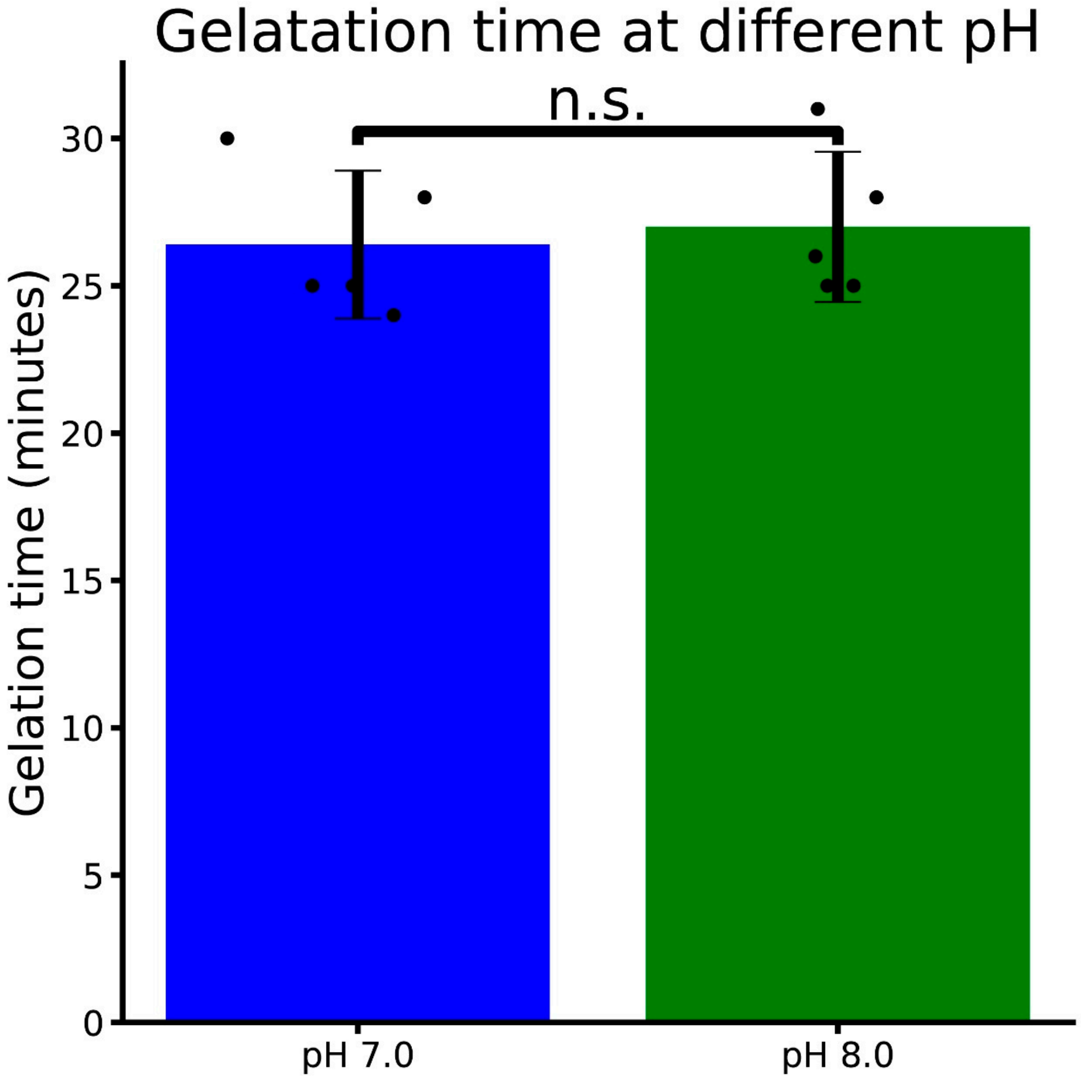

Figure S8. Gelation Time of Protein Solution at Different pH Levels. The chart reveals that the gelation time does not significantly change (n.s., not significant) with an increase in pH from 7.0 to 8.0. The error bars represent standard deviation. Error bars depict the standard deviation.

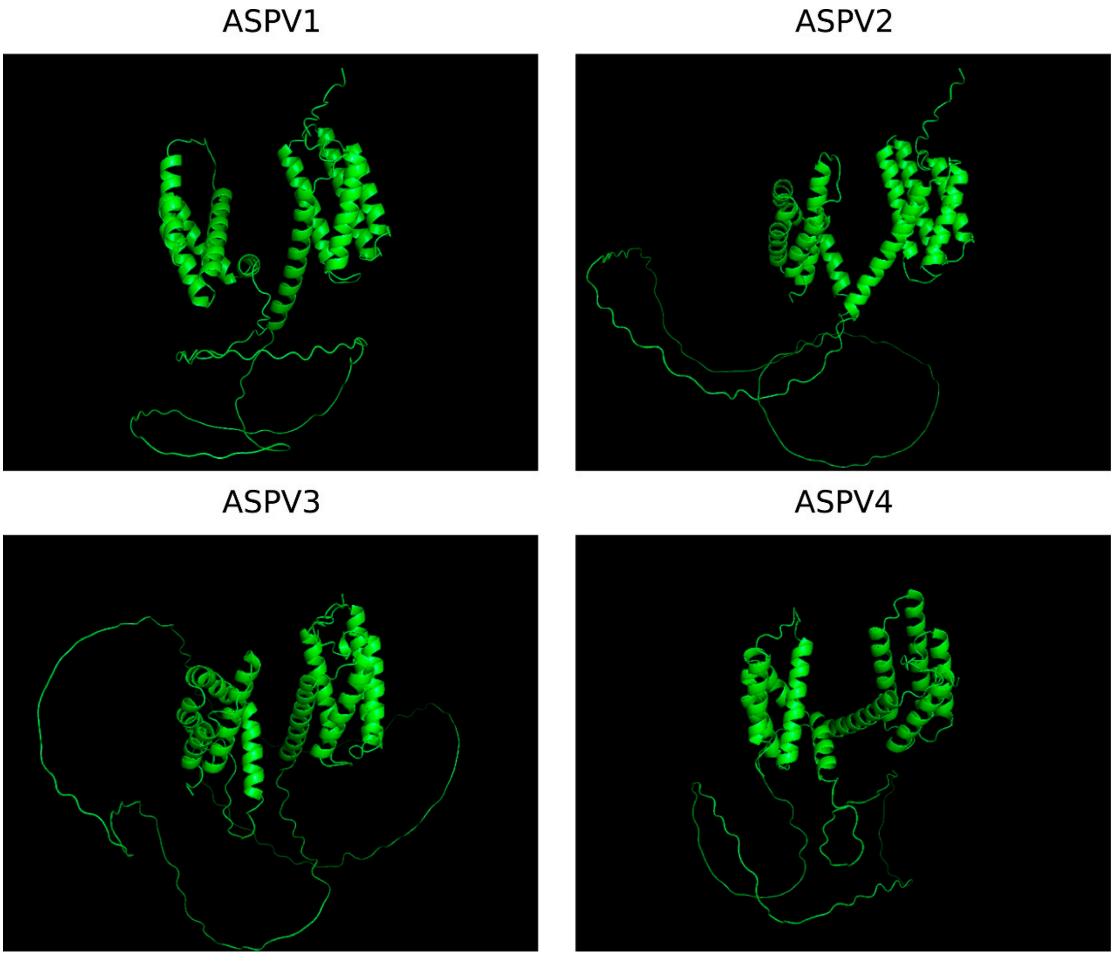

Figure S9. Predicted 3D Structures of ASPs by AlphaFold2. Each panel corresponds to a ASP, showing the intricate folds and coils characteristic of protein tertiary structures.

Seq 1: pASPV1

GGGGAATTGTGAGCGGATAACAATTCCCCTCTAGAAATAATTTTGTTTAACTTTAAGAAGGAGAT
ATACCATGCATCATCACCACCACCACCACCATTCACATACCACTCCGTGGACCAATCCGGGTCT
GGCGGAGAACTTCATGAACTCCTTTATGCAGGGCCTGAGCAGCATGCCGGGTTTCACCGCAAG
CCAGCTGGACGACATGAGCACGATTGCGCAAAGCATGGTTCAGTCGATCCAGAGCTTGGCGGC
CCAGGGTCGCACCTCCCCGAATAAGCTGCAGGCACTGAACATGGCGTTCGCCAGCTCCATGGC
AGAGATCGCGGCGTCTGAAGAAGGTGGTGGTAGTTTGTCTACGAAAACCAGCTCTATCGCGTCT
GCTATGAGCAACGCATTCCTGCAGACCACTGGTGTGGTGAACCAGCCATTTATCAATGAAATCA
CCCAGCTCGTTAGCATGTTTGCACAAGCTGGCATGAATGATGTTAGCGCGGCTAGCCAGGGTGG
GTTTTCCGGCCAAGGGCAAGGGGGGTTTGGTCCAGGCGCTGGGTCCAGCGCAGCTGCAGCAG
CAGCAGCTGCCGCGGCTGCACGGCAAGGTGGGCAAGGCCAAGGGGGTTTTGGTCAGGGGGCA
GGTGGCAACGCCGCGGCAGCAGCTGCAGCTGCCGCAGCTGCGGCCGCTGCACAACAAGGTGG
GCAAGGGGGTTTTTCGGGGCGCGGCCAAGGCGGTTTCGGGCCGGGTGCCGGGAGCAGCACTA
GTGTTACCAGCGGCGGCTACGGTTACGGCACCTCCGCGGCGGCTGGTGCGGGCGTCGCAGCG
GGTAGCTATGCCGGCGCGGTTAATCGTCTGAGCTCAGCGGAGGCAGCGTCTCGTGTTTCCTCCA
ACATCGCCGCGATTGCCTCGGGCGGCGCAAGCGCTTTACCGTCTGTTATTAGCAACATTTATAG
CGGCGTCGTGGCATCGGGCGTGAGCTCTAACGAGGCCCTGATTCAGGCACTTTTGGAACTGCT
GTCTGCCTTGGTGCACGTGCTGAGCTCAGCTAGTATCGGTAACGTGAGCTCCGTGGGTGTCGAC
AGCACGCTGAATGTAGTTCAGGATTCCGTGGGCCAATATGTTGGTAAAAAGTAAGGACTCGTCT
ACTAGCGCAGCTTAATTAACCTAGGCTGCTGCCACCGCTGAGCAATAACTAGCATAACCCCTTG
GGGCCTCTAAACGGGTCTTGAGGGGTTTTTTGCTGAAAGGAGGAACTATATCCGGATTGGCGAA
TGGGACGCGCCCTGTAGCGGCGCATTAAGCGCGGCGGGTGTGGTGGTTACGCGCAGCGTGAC
CGCTACACTTGCCAGCGCCCTAGCGCCCGCTCCTTTCGCTTTCTTCCCTTCCTTTCTCGCCACGTT
CGCCGGCTTTCCCCGTCAAGCTCTAAATCGGGGGCTCCCTTTAGGGTTCCGATTTAGTGCTTTAC
GGCACCTCGACCCCAAAAAACTTGATTAGGGTGATGGTTCACGTAGTGGGCCATCGCCCTGATA
GACGGTTTTTCGCCCTTTGACGTTGGAGTCCACGTTCTTTAATAGTGGACTCTTGTTCCAAACTGG
AACAACACTCAACCCTATCTCGGTCTATTCTTTTGATTTATAAGGGATTTTGCCGATTTCGGCCTA
TTGGTTAAAAAATGAGCTGATTTAACAAAAATTTAACGCGAATTTTAACAAAATATTAACGTTTA
CAATTTCTGGCGGCACGATGGCATGAGATTATCAAAAAGGATCTTCACCTAGATCCTTTTAAATT
AAAAATGAAGTTTTAAATCAATCTAAAGTATATATGAGTAAACTTGGTCTGACAGTTACCAATGC
TTAATCAGTGAGGCACCTATCTCAGCGATCTGTCTATTTCGTTCATCCATAGTTGCCTGACTCCCC
GTCGTGTAGATAACTACGATACGGGAGGGCTTACCATCTGGCCCCAGTGCTGCAATGATACCGC
GAGACCCACGCTCACCGGCTCCAGATTTATCAGCAATAAACCAGCCAGCCGGAAGGGCCGAGC
GCAGAAGTGGTCCTGCAACTTTATCCGCCTCCATCCAGTCTATTAATTGTTGCCGGGAAGCTAG
AGTAAGTAGTTCGCCAGTTAATAGTTTGCGCAACGTTGTTGCCATTGCTACAGGCATCGTGGTGT
CACGCTCGTCGTTTGGTATGGCTTCATTCAGCTCCGGTTCCCAACGATCAAGGCGAGTTACATG
ATCCCCCATGTTGTGCAAAAAAGCGGTTAGCTCCTTCGGTCCTCCGATCGTTGTCAGAAGTAAG
TTGGCCGCAGTGTTATCACTCATGGTTATGGCAGCACTGCATAATTCTCTTACTGTCATGCCATC
CGTAAGATGCTTTTCTGTGACTGGTGAGTACTCAACCAAGTCATTCTGAGAATAGTGTATGCGGC
GACCGAGTTGCTCTTGCCCGGCGTCAATACGGGATAATACCGCGCCACATAGCAGAACTTTAAA
AGTGCTCATCATTGGAAAACGTTCTTCGGGGCGAAAACTCTCAAGGATCTTACCGCTGTTGAGA
TCCAGTTCGATGTAACCCACTCGTGCACCCAACTGATCTTCAGCATCTTTTACTTTCACCAGCGTT
TCTGGGTGAGCAAAAACAGGAAGGCAAAATGCCGCAAAAAAGGGAATAAGGGCGACACGGAA
ATGTTGAATACTCATACTCTTCCTTTTTCAATCATGATTGAAGCATTTATCAGGGTTATTGTCTCAT

GAGCGGATACATATTTGAATGTATTTAGAAAAATAAACAAATAGGTCATGACCAAAATCCCTTA
ACGTGAGTTTTCGTTCCACTGAGCGTCAGACCCCGTAGAAAAGATCAAAGGATCTTCTTGAGAT
CCTTTTTTTCTGCGCGTAATCTGCTGCTTGCAAACAAAAAAACCACCGCTACCAGCGGTGGTTTG
TTTGCCGGATCAAGAGCTACCAACTCTTTTTCCGAAGGTAACTGGCTTCAGCAGAGCGCAGATA
CCAAATACTGTCCTTCTAGTGTAGCCGTAGTTAGGCCACCACTTCAAGAACTCTGTAGCACCGC
CTACATACCTCGCTCTGCTAATCCTGTTACCAGTGGCTGCTGCCAGTGGCGATAAGTCGTGTCTT
ACCGGGTTGGACTCAAGACGATAGTTACCGGATAAGGCGCAGCGGTCGGGCTGAACGGGGGG
TTCGTGCACACAGCCCAGCTTGGAGCGAACGACCTACACCGAACTGAGATACCTACAGCGTGA
GCTATGAGAAAGCGCCACGCTTCCCGAAGGGAGAAAGGCGGACAGGTATCCGGTAAGCGGCA
GGGTCGGAACAGGAGAGCGCACGAGGGAGCTTCCAGGGGGAAACGCCTGGTATCTTTATAGTC
CTGTCGGGTTTCGCCACCTCTGACTTGAGCGTCGATTTTTGTGATGCTCGTCAGGGGGGCGGAG
CCTATGGAAAAACGCCAGCAACGCGGCCTTTTTACGGTTCCTGGCCTTTTGCTGGCCTTTTGCTC
ACATGTTCTTTCCTGCGTTATCCCCTGATTCTGTGGATAACCGTATTACCGCCTTTGAGTGAGCTG
ATACCGCTCGCCGCAGCCGAACGACCGAGCGCAGCGAGTCAGTGAGCGAGGAAGCGGAAGA
GCGCCTGATGCGGTATTTTCTCCTTACGCATCTGTGCGGTATTTCACACCGCATATATGGTGCAC
TCTCAGTACAATCTGCTCTGATGCCGCATAGTTAAGCCAGTATACACTCCGCTATCGCTACGTGA
CTGGGTCATGGCTGCGCCCCGACACCCGCCAACACCCGCTGACGCGCCCTGACGGGCTTGTCT
GCTCCCGGCATCCGCTTACAGACAAGCTGTGACCGTCTCCGGGAGCTGCATGTGTCAGAGGTTT
TCACCGTCATCACCGAAACGCGCGAGGCAGCTGCGGTAAAGCTCATCAGCGTGGTCGTGAAGC
GATTCACAGATGTCTGCCTGTTCATCCGCGTCCAGCTCGTTGAGTTTCTCCAGAAGCGTTAATGT
CTGGCTTCTGATAAAGCGGGCCATGTTAAGGGCGGTTTTTTCCTGTTTGGTCACTGATGCCTCCG
TGTAAGGGGGATTTCTGTTCATGGGGGTAATGATACCGATGAAACGAGAGAGGATGCTCACGA
TACGGGTTACTGATGATGAACATGCCCGGTTACTGGAACGTTGTGAGGGTAAACAACTGGCGGT
ATGGATGCGGCGGGACCAGAGAAAAATCACTCAGGGTCAATGCCAGCGCTTCGTTAATACAGA
TGTAGGTGTTCCACAGGGTAGCCAGCAGCATCCTGCGATGCAGATCCGGAACATAATGGTGCA
GGGCGCTGACTTCCGCGTTTCCAGACTTTACGAAACACGGAAACCGAAGACCATTCATGTTGTT
GCTCAGGTCGCAGACGTTTTGCAGCAGCAGTCGCTTCACGTTCGCTCGCGTATCGGTGATTCATT
CTGCTAACCAGTAAGGCAACCCCGCCAGCCTAGCCGGGTCCTCAACGACAGGAGCACGATCAT
GCTAGTCATGCCCCGCGCCCACCGGAAGGAGCTGACTGGGTTGAAGGCTCTCAAGGGCATCGG
TCGAGATCCCGGTGCCTAATGAGTGAGCTAACTTACATTAATTGCGTTGCGCTCACTGCCCGCTT
TCCAGTCGGGAAACCTGTCGTGCCAGCTGCATTAATGAATCGGCCAACGCGCGGGGAGAGGCG
GTTTGCGTATTGGGCGCCAGGGTGGTTTTTCTTTTCACCAGTGAGACGGGCAACAGCTGATTGC
CCTTCACCGCCTGGCCCTGAGAGAGTTGCAGCAAGCGGTCCACGCTGGTTTGCCCCAGCAGGC
GAAAATCCTGTTTGATGGTGGTTAACGGCGGGATATAACATGAGCTGTCTTCGGTATCGTCGTAT
CCCACTACCGAGATGTCCGCACCAACGCGCAGCCCGGACTCGGTAATGGCGCGCATTGCGCCC
AGCGCCATCTGATCGTTGGCAACCAGCATCGCAGTGGGAACGATGCCCTCATTCAGCATTTGCA
TGGTTTGTTGAAAACCGGACATGGCACTCCAGTCGCCTTCCCGTTCCGCTATCGGCTGAATTTGA
TTGCGAGTGAGATATTTATGCCAGCCAGCCAGACGCAGACGCGCCGAGACAGAACTTAATGGG
CCCGCTAACAGCGCGATTTGCTGGTGACCCAATGCGACCAGATGCTCCACGCCCAGTCGCGTA
CCGTCTTCATGGGAGAAAATAATACTGTTGATGGGTGTCTGGTCAGAGACATCAAGAAATAACG
CCGGAACATTAGTGCAGGCAGCTTCCACAGCAATGGCATCCTGGTCATCCAGCGGATAGTTAAT
GATCAGCCCACTGACGCGTTGCGCGAGAAGATTGTGCACCGCCGCTTTACAGGCTTCGACGCC
GCTTCGTTCTACCATCGACACCACCACGCTGGCACCCAGTTGATCGGCGCGAGATTTAATCGCC
GCGACAATTTGCGACGGCGCGTGCAGGGCCAGACTGGAGGTGGCAACGCCAATCAGCAACGA

CTGTTTGCCCGCCAGTTGTTGTGCCACGCGGTTGGGAATGTAATTCAGCTCCGCCATCGCCGCTT
CCACTTTTTCCCGCGTTTTCGCAGAAACGTGGCTGGCCTGGTTCACCACGCGGGAAACGGTCTG
ATAAGAGACACCGGCATACTCTGCGACATCGTATAACGTTACTGGTTTCACATTCACCACCCTG
AATTGACTCTCTTCCGGGCGCTATCATGCCATACCGCGAAAGGTTTTGCGCCATTCGATGGTGTC
CGGGATCTCGACGCTCTCCCTTATGCGACTCCTGCATTAGGAAGCAGCCCAGTAGTAGGTTGAG
GCCGTTGAGCACCGCCGCCGCAAGGAATGGTGCATGCAAGGAGATGGCGCCCAACAGTCCCC
CGGCCACGGGGCCTGCCACCATACCCACGCCGAAACAAGCGCTCATGAGCCCGAAGTGGCGA
GCCCGATCTTCCCCATCGGTGATGTCGGCGATATAGGCGCCAGCAACCGCACCTGTGGCGCCG
GTGATGCCGGCCACGATGCGTCCGGCGTAGAGGATCGAGATCGATCTCGATCCCGCGAAATTA
ATACGACTCACTATA

Seq 2: pASPV2

GGGGAATTGTGAGCGGATAACAATTCCCCTCTAGAAATAATTTTGTTTAACTTTAAGAAGGA
GATATACCATGCATCATCACCACCACCACCACCATTCACATACCACTCCGTGGACCAATCC
GGGTCTGGCGGAGAACTTCATGAACTCCTTTATGCAGGGCCTGAGCAGCATGCCGGGTTTC
ACCGCAAGCCAGCTGGACGACATGAGCACGATTGCGCAAAGCATGGTTCAGTCGATCCAG
AGCTTGGCGGCCCAGGGTCGCACCTCCCCGAATAAGCTGCAGGCACTGAACATGGCGTTC
GCCAGCTCCATGGCAGAGATCGCGGCGTCTGAAGAAGGTGGTGGTAGTTTGTCTACGAAA
ACCAGCTCTATCGCGTCTGCTATGAGCAACGCATTCCTGCAGACCACTGGTGTGGTGAACC
AGCCATTTATCAATGAAATCACCCAGCTCGTTAGCATGTTTGCACAAGCTGGCATGAATGAT
GTTAGCGCGGCTAGCGGTTATGGGGGTCTGGGTGGCCAAGGGGCGGGACAAGGTGCCGG
TGCAGCTGCAGCGGCCGCCGGTGGCGCAGGTCAAGGGTACGGCGGGTTAGGTGGGCAAG
GGGCAGGGGCAGCAGCCGCTGCGGCGGGTGGGGCTGGTCAAGGTGGCTATGGTGGTTTG
GGTGGTCAAGGTGCCGGTCAAGGTGGCGGTGCGGCCGCAGCAGCGGCAGGCGGCGCAG
GTCAGGGCGGTTACGGTGGATTGGGCGGTCAAGGAGCAGGTCAAGGTGCAGGTGCAGCC
GCAGCCGCAGCAGGTGGTGCAGGTCAAGGCACTAGTGTTACCAGCGGCGGCTACGGTTAC
GGCACCTCCGCGGCGGCTGGTGCGGGCGTCGCAGCGGGTAGCTATGCCGGCGCGGTTAAT
CGTCTGAGCTCAGCGGAGGCAGCGTCTCGTGTTTCCTCCAACATCGCCGCGATTGCCTCGG
GCGGCGCAAGCGCTTTACCGTCTGTTATTAGCAACATTTATAGCGGCGTCGTGGCATCGGG
CGTGAGCTCTAACGAGGCCCTGATTCAGGCACTTTTGGAACTGCTGTCTGCCTTGGTGCAC
GTGCTGAGCTCAGCTAGTATCGGTAACGTGAGCTCCGTGGGTGTCGACAGCACGCTGAAT
GTAGTTCAGGATTCCGTGGGCCAATATGTTGGTAAAAAGTAAGGACTCGTCTACTAGCGCA
GCTTAATTAACCTAGGCTGCTGCCACCGCTGAGCAATAACTAGCATAACCCCTTGGGGCCT
CTAAACGGGTCTTGAGGGGTTTTTTGCTGAAAGGAGGAACTATATCCGGATTGGCGAATGG
GACGCGCCCTGTAGCGGCGCATTAAGCGCGGCGGGTGTGGTGGTTACGCGCAGCGTGACC
GCTACACTTGCCAGCGCCCTAGCGCCCGCTCCTTTCGCTTTCTTCCCTTCCTTTCTCGCCAC
GTTCGCCGGCTTTCCCCGTCAAGCTCTAAATCGGGGGCTCCCTTTAGGGTTCCGATTTAGTG
CTTTACGGCACCTCGACCCCAAAAAACTTGATTAGGGTGATGGTTCACGTAGTGGGCCATC
GCCCTGATAGACGGTTTTTCGCCCTTTGACGTTGGAGTCCACGTTCTTTAATAGTGGACTCTT
GTTCCAAACTGGAACAACACTCAACCCTATCTCGGTCTATTCTTTTGATTTATAAGGGATTTT
GCCGATTTCGGCCTATTGGTTAAAAAATGAGCTGATTTAACAAAAATTTAACGCGAATTTTA
ACAAAATATTAACGTTTACAATTTCTGGCGGCACGATGGCATGAGATTATCAAAAAGGATCT
TCACCTAGATCCTTTTAAATTAAAAATGAAGTTTTAAATCAATCTAAAGTATATATGAGTAAA
CTTGGTCTGACAGTTACCAATGCTTAATCAGTGAGGCACCTATCTCAGCGATCTGTCTATTT
CGTTCATCCATAGTTGCCTGACTCCCCGTCGTGTAGATAACTACGATACGGGAGGGCTTAC
CATCTGGCCCCAGTGCTGCAATGATACCGCGAGACCCACGCTCACCGGCTCCAGATTTATC
AGCAATAAACCAGCCAGCCGGAAGGGCCGAGCGCAGAAGTGGTCCTGCAACTTTATCCGC
CTCCATCCAGTCTATTAATTGTTGCCGGGAAGCTAGAGTAAGTAGTTCGCCAGTTAATAGTT
TGCGCAACGTTGTTGCCATTGCTACAGGCATCGTGGTGTCACGCTCGTCGTTTGGTATGGCT
TCATTCAGCTCCGGTTCCCAACGATCAAGGCGAGTTACATGATCCCCCATGTTGTGCAAAA
AAGCGGTTAGCTCCTTCGGTCCTCCGATCGTTGTCAGAAGTAAGTTGGCCGCAGTGTTATC
ACTCATGGTTATGGCAGCACTGCATAATTCTCTTACTGTCATGCCATCCGTAAGATGCTTTTC
TGTGACTGGTGAGTACTCAACCAAGTCATTCTGAGAATAGTGTATGCGGCGACCGAGTTGC
TCTTGCCCGGCGTCAATACGGGATAATACCGCGCCACATAGCAGAACTTTAAAAGTGCTCA
TCATTGGAAAACGTTCTTCGGGGCGAAAACTCTCAAGGATCTTACCGCTGTTGAGATCCAG

TTCGATGTAACCCACTCGTGCACCCAACTGATCTTCAGCATCTTTTACTTTCACCAGCGTTTC
TGGGTGAGCAAAAACAGGAAGGCAAAATGCCGCAAAAAAGGGAATAAGGGCGACACGGA
AATGTTGAATACTCATACTCTTCCTTTTTCAATCATGATTGAAGCATTTATCAGGGTTATTGTC
TCATGAGCGGATACATATTTGAATGTATTTAGAAAAATAAACAAATAGGTCATGACCAAAAT
CCCTTAACGTGAGTTTTCGTTCCACTGAGCGTCAGACCCCGTAGAAAAGATCAAAGGATCT
TCTTGAGATCCTTTTTTTCTGCGCGTAATCTGCTGCTTGCAAACAAAAAAACCACCGCTACC
AGCGGTGGTTTGTTTGCCGGATCAAGAGCTACCAACTCTTTTTCCGAAGGTAACTGGCTTCA
GCAGAGCGCAGATACCAAATACTGTCCTTCTAGTGTAGCCGTAGTTAGGCCACCACTTCAA
GAACTCTGTAGCACCGCCTACATACCTCGCTCTGCTAATCCTGTTACCAGTGGCTGCTGCCA
GTGGCGATAAGTCGTGTCTTACCGGGTTGGACTCAAGACGATAGTTACCGGATAAGGCGC
AGCGGTCGGGCTGAACGGGGGGTTCGTGCACACAGCCCAGCTTGGAGCGAACGACCTAC
ACCGAACTGAGATACCTACAGCGTGAGCTATGAGAAAGCGCCACGCTTCCCGAAGGGAGA
AAGGCGGACAGGTATCCGGTAAGCGGCAGGGTCGGAACAGGAGAGCGCACGAGGGAGC
TTCCAGGGGGAAACGCCTGGTATCTTTATAGTCCTGTCGGGTTTCGCCACCTCTGACTTGAG
CGTCGATTTTTGTGATGCTCGTCAGGGGGGCGGAGCCTATGGAAAAACGCCAGCAACGCG
GCCTTTTTACGGTTCCTGGCCTTTTGCTGGCCTTTTGCTCACATGTTCTTTCCTGCGTTATCCC
CTGATTCTGTGGATAACCGTATTACCGCCTTTGAGTGAGCTGATACCGCTCGCCGCAGCCG
AACGACCGAGCGCAGCGAGTCAGTGAGCGAGGAAGCGGAAGAGCGCCTGATGCGGTATT
TTCTCCTTACGCATCTGTGCGGTATTTCACACCGCATATATGGTGCACTCTCAGTACAATCTG
CTCTGATGCCGCATAGTTAAGCCAGTATACACTCCGCTATCGCTACGTGACTGGGTCATGG
CTGCGCCCCGACACCCGCCAACACCCGCTGACGCGCCCTGACGGGCTTGTCTGCTCCCGG
CATCCGCTTACAGACAAGCTGTGACCGTCTCCGGGAGCTGCATGTGTCAGAGGTTTTCACC
GTCATCACCGAAACGCGCGAGGCAGCTGCGGTAAAGCTCATCAGCGTGGTCGTGAAGCGA
TTCACAGATGTCTGCCTGTTCATCCGCGTCCAGCTCGTTGAGTTTCTCCAGAAGCGTTAATG
TCTGGCTTCTGATAAAGCGGGCCATGTTAAGGGCGGTTTTTTCCTGTTTGGTCACTGATGCC
TCCGTGTAAGGGGGATTTCTGTTCATGGGGGTAATGATACCGATGAAACGAGAGAGGATG
CTCACGATACGGGTTACTGATGATGAACATGCCCGGTTACTGGAACGTTGTGAGGGTAAAC
AACTGGCGGTATGGATGCGGCGGGACCAGAGAAAAATCACTCAGGGTCAATGCCAGCGCT
TCGTTAATACAGATGTAGGTGTTCCACAGGGTAGCCAGCAGCATCCTGCGATGCAGATCCG
GAACATAATGGTGCAGGGCGCTGACTTCCGCGTTTCCAGACTTTACGAAACACGGAAACC
GAAGACCATTCATGTTGTTGCTCAGGTCGCAGACGTTTTGCAGCAGCAGTCGCTTCACGTTC
GCTCGCGTATCGGTGATTCATTCTGCTAACCAGTAAGGCAACCCCGCCAGCCTAGCCGGGT
CCTCAACGACAGGAGCACGATCATGCTAGTCATGCCCCGCGCCCACCGGAAGGAGCTGAC
TGGGTTGAAGGCTCTCAAGGGCATCGGTCGAGATCCCGGTGCCTAATGAGTGAGCTAACTT
ACATTAATTGCGTTGCGCTCACTGCCCGCTTTCCAGTCGGGAAACCTGTCGTGCCAGCTGC
ATTAATGAATCGGCCAACGCGCGGGGAGAGGCGGTTTGCGTATTGGGCGCCAGGGTGGTT
TTTCTTTTCACCAGTGAGACGGGCAACAGCTGATTGCCCTTCACCGCCTGGCCCTGAGAGA
GTTGCAGCAAGCGGTCCACGCTGGTTTGCCCCAGCAGGCGAAAATCCTGTTTGATGGTGGT
TAACGGCGGGATATAACATGAGCTGTCTTCGGTATCGTCGTATCCCACTACCGAGATGTCC
GCACCAACGCGCAGCCCGGACTCGGTAATGGCGCGCATTGCGCCCAGCGCCATCTGATCG
TTGGCAACCAGCATCGCAGTGGGAACGATGCCCTCATTCAGCATTTGCATGGTTTGTTGAA
AACCGGACATGGCACTCCAGTCGCCTTCCCGTTCCGCTATCGGCTGAATTTGATTGCGAGT
GAGATATTTATGCCAGCCAGCCAGACGCAGACGCGCCGAGACAGAACTTAATGGGCCCGC
TAACAGCGCGATTTGCTGGTGACCCAATGCGACCAGATGCTCCACGCCCAGTCGCGTACC

GTCTTCATGGGAGAAAATAATACTGTTGATGGGTGTCTGGTCAGAGACATCAAGAAATAAC
GCCGGAACATTAGTGCAGGCAGCTTCCACAGCAATGGCATCCTGGTCATCCAGCGGATAG
TTAATGATCAGCCCACTGACGCGTTGCGCGAGAAGATTGTGCACCGCCGCTTTACAGGCTT
CGACGCCGCTTCGTTCTACCATCGACACCACCACGCTGGCACCCAGTTGATCGGCGCGAG
ATTTAATCGCCGCGACAATTTGCGACGGCGCGTGCAGGGCCAGACTGGAGGTGGCAACGC
CAATCAGCAACGACTGTTTGCCCGCCAGTTGTTGTGCCACGCGGTTGGGAATGTAATTCAG
CTCCGCCATCGCCGCTTCCACTTTTTCCCGCGTTTTCGCAGAAACGTGGCTGGCCTGGTTCA
CCACGCGGGAAACGGTCTGATAAGAGACACCGGCATACTCTGCGACATCGTATAACGTTA
CTGGTTTCACATTCACCACCCTGAATTGACTCTCTTCCGGGCGCTATCATGCCATACCGCGA
AAGGTTTTGCGCCATTCGATGGTGTCCGGGATCTCGACGCTCTCCCTTATGCGACTCCTGCA
TTAGGAAGCAGCCCAGTAGTAGGTTGAGGCCGTTGAGCACCGCCGCCGCAAGGAATGGTG
CATGCAAGGAGATGGCGCCCAACAGTCCCCCGGCCACGGGGCCTGCCACCATACCCACG
CCGAAACAAGCGCTCATGAGCCCGAAGTGGCGAGCCCGATCTTCCCCATCGGTGATGTCG
GCGATATAGGCGCCAGCAACCGCACCTGTGGCGCCGGTGATGCCGGCCACGATGCGTCCG
GCGTAGAGGATCGAGATCGATCTCGATCCCGCGAAATTAATACGACTCACTATA

Seq 3: pASPV3

GGGGAATTGTGAGCGGATAACAATTCCCCTCTAGAAATAATTTTGTTTAACTTTAAGAAGGA
GATATACCATGCATCATCACCACCACCACCACCATTCACATACCACTCCGTGGACCAATCC
GGGTCTGGCGGAGAACTTCATGAACTCCTTTATGCAGGGCCTGAGCAGCATGCCGGGTTTC
ACCGCAAGCCAGCTGGACGACATGAGCACGATTGCGCAAAGCATGGTTCAGTCGATCCAG
AGCTTGGCGGCCCAGGGTCGCACCTCCCCGAATAAGCTGCAGGCACTGAACATGGCGTTC
GCCAGCTCCATGGCAGAGATCGCGGCGTCTGAAGAAGGTGGTGGTAGTTTGTCTACGAAA
ACCAGCTCTATCGCGTCTGCTATGAGCAACGCATTCCTGCAGACCACTGGTGTGGTGAACC
AGCCATTTATCAATGAAATCACCCAGCTCGTTAGCATGTTTGCACAAGCTGGCATGAATGAT
GTTAGCGCGGCTAGCTATGGTCCGTCAGGCCCGGGGAGCGCCGCAGCGGCAGCCGCCGC
AGCAGGTGCAGGACCTGGAGGCTATGGCCCGGGTCAACAAGGTCCTGGCCAGCAAGGCC
CAGGGGGTTACGGGCCTTCTGGTCCGGGGAGTGCAGCAGCTGCTGCGGCAGCCGCCGGG
GCCGGTCCTGGTGGCTACGGTCCTGGTCAACAAGGTCCAGGTCAGCAAGGCCCTGGGCAA
CAGGGGCCGGGGGGCTATGGCCCTTCAGGGCCGGGGTCCGCGGCTGCTGCCGCTGCAGC
CGCCGGTGCCGGTCCAGGTGGTTATGGTCCGGGCCAACAGGGGCCAGGACAACAGGGTC
CAGGACAACAAGGTCCGGGTCAACAGGGTCCGGGCGGCTATGGTCCGTCCGGTCCAGGTT
CAGCCGCAGCTGCTGCAGCAGCCGCTGGTGCAGGTCCTGGTGGCTATGGGCCGGGCCAG
CAAGGGCCAGGGCAACAAGGGCCTGGGGGCACTAGTGTTACCAGCGGCGGCTACGGTTA
CGGCACCTCCGCGGCGGCTGGTGCGGGCGTCGCAGCGGGTAGCTATGCCGGCGCGGTTA
ATCGTCTGAGCTCAGCGGAGGCAGCGTCTCGTGTTTCCTCCAACATCGCCGCGATTGCCTC
GGGCGGCGCAAGCGCTTTACCGTCTGTTATTAGCAACATTTATAGCGGCGTCGTGGCATCG
GGCGTGAGCTCTAACGAGGCCCTGATTCAGGCACTTTTGGAACTGCTGTCTGCCTTGGTGC
ACGTGCTGAGCTCAGCTAGTATCGGTAACGTGAGCTCCGTGGGTGTCGACAGCACGCTGA
ATGTAGTTCAGGATTCCGTGGGCCAATATGTTGGTAAAAAGTAAGGACTCGTCTACTAGCG
CAGCTTAATTAACCTAGGCTGCTGCCACCGCTGAGCAATAACTAGCATAACCCCTTGGGGC
CTCTAAACGGGTCTTGAGGGGTTTTTTGCTGAAAGGAGGAACTATATCCGGATTGGCGAAT
GGGACGCGCCCTGTAGCGGCGCATTAAGCGCGGCGGGTGTGGTGGTTACGCGCAGCGTG
ACCGCTACACTTGCCAGCGCCCTAGCGCCCGCTCCTTTCGCTTTCTTCCCTTCCTTTCTCGCC
ACGTTCGCCGGCTTTCCCCGTCAAGCTCTAAATCGGGGGCTCCCTTTAGGGTTCCGATTTAG
TGCTTTACGGCACCTCGACCCCAAAAAACTTGATTAGGGTGATGGTTCACGTAGTGGGCCA
TCGCCCTGATAGACGGTTTTTCGCCCTTTGACGTTGGAGTCCACGTTCTTTAATAGTGGACT
CTTGTTCCAAACTGGAACAACACTCAACCCTATCTCGGTCTATTCTTTTGATTTATAAGGGAT
TTTGCCGATTTCGGCCTATTGGTTAAAAAATGAGCTGATTTAACAAAAATTTAACGCGAATT
TTAACAAAATATTAACGTTTACAATTTCTGGCGGCACGATGGCATGAGATTATCAAAAAGG
ATCTTCACCTAGATCCTTTTAAATTAAAAATGAAGTTTTAAATCAATCTAAAGTATATATGAG
TAAACTTGGTCTGACAGTTACCAATGCTTAATCAGTGAGGCACCTATCTCAGCGATCTGTCT
ATTTCGTTCATCCATAGTTGCCTGACTCCCCGTCGTGTAGATAACTACGATACGGGAGGGCT
TACCATCTGGCCCCAGTGCTGCAATGATACCGCGAGACCCACGCTCACCGGCTCCAGATTT
ATCAGCAATAAACCAGCCAGCCGGAAGGGCCGAGCGCAGAAGTGGTCCTGCAACTTTATC
CGCCTCCATCCAGTCTATTAATTGTTGCCGGGAAGCTAGAGTAAGTAGTTCGCCAGTTAATA
GTTTGCGCAACGTTGTTGCCATTGCTACAGGCATCGTGGTGTCACGCTCGTCGTTTGGTATG
GCTTCATTCAGCTCCGGTTCCCAACGATCAAGGCGAGTTACATGATCCCCCATGTTGTGCA
AAAAAGCGGTTAGCTCCTTCGGTCCTCCGATCGTTGTCAGAAGTAAGTTGGCCGCAGTGTT
ATCACTCATGGTTATGGCAGCACTGCATAATTCTCTTACTGTCATGCCATCCGTAAGATGCT

TTTCTGTGACTGGTGAGTACTCAACCAAGTCATTCTGAGAATAGTGTATGCGGCGACCGAG
TTGCTCTTGCCCGGCGTCAATACGGGATAATACCGCGCCACATAGCAGAACTTTAAAAGTG
CTCATCATTGGAAAACGTTCTTCGGGGCGAAAACTCTCAAGGATCTTACCGCTGTTGAGAT
CCAGTTCGATGTAACCCACTCGTGCACCCAACTGATCTTCAGCATCTTTTACTTTCACCAGC
GTTTCTGGGTGAGCAAAAACAGGAAGGCAAAATGCCGCAAAAAAGGGAATAAGGGCGAC
ACGGAAATGTTGAATACTCATACTCTTCCTTTTTCAATCATGATTGAAGCATTTATCAGGGTT
ATTGTCTCATGAGCGGATACATATTTGAATGTATTTAGAAAAATAAACAAATAGGTCATGAC
CAAAATCCCTTAACGTGAGTTTTCGTTCCACTGAGCGTCAGACCCCGTAGAAAAGATCAAA
GGATCTTCTTGAGATCCTTTTTTTCTGCGCGTAATCTGCTGCTTGCAAACAAAAAAACCACC
GCTACCAGCGGTGGTTTGTTTGCCGGATCAAGAGCTACCAACTCTTTTTCCGAAGGTAACTG
GCTTCAGCAGAGCGCAGATACCAAATACTGTCCTTCTAGTGTAGCCGTAGTTAGGCCACCA
CTTCAAGAACTCTGTAGCACCGCCTACATACCTCGCTCTGCTAATCCTGTTACCAGTGGCTG
CTGCCAGTGGCGATAAGTCGTGTCTTACCGGGTTGGACTCAAGACGATAGTTACCGGATAA
GGCGCAGCGGTCGGGCTGAACGGGGGGTTCGTGCACACAGCCCAGCTTGGAGCGAACGA
CCTACACCGAACTGAGATACCTACAGCGTGAGCTATGAGAAAGCGCCACGCTTCCCGAAG
GGAGAAAGGCGGACAGGTATCCGGTAAGCGGCAGGGTCGGAACAGGAGAGCGCACGAG
GGAGCTTCCAGGGGGAAACGCCTGGTATCTTTATAGTCCTGTCGGGTTTCGCCACCTCTGA
CTTGAGCGTCGATTTTTGTGATGCTCGTCAGGGGGGCGGAGCCTATGGAAAAACGCCAGC
AACGCGGCCTTTTTACGGTTCCTGGCCTTTTGCTGGCCTTTTGCTCACATGTTCTTTCCTGCG
TTATCCCCTGATTCTGTGGATAACCGTATTACCGCCTTTGAGTGAGCTGATACCGCTCGCCG
CAGCCGAACGACCGAGCGCAGCGAGTCAGTGAGCGAGGAAGCGGAAGAGCGCCTGATGC
GGTATTTTCTCCTTACGCATCTGTGCGGTATTTCACACCGCATATATGGTGCACTCTCAGTAC
AATCTGCTCTGATGCCGCATAGTTAAGCCAGTATACACTCCGCTATCGCTACGTGACTGGGT
CATGGCTGCGCCCCGACACCCGCCAACACCCGCTGACGCGCCCTGACGGGCTTGTCTGCT
CCCGGCATCCGCTTACAGACAAGCTGTGACCGTCTCCGGGAGCTGCATGTGTCAGAGGTTT
TCACCGTCATCACCGAAACGCGCGAGGCAGCTGCGGTAAAGCTCATCAGCGTGGTCGTGA
AGCGATTCACAGATGTCTGCCTGTTCATCCGCGTCCAGCTCGTTGAGTTTCTCCAGAAGCGT
TAATGTCTGGCTTCTGATAAAGCGGGCCATGTTAAGGGCGGTTTTTTCCTGTTTGGTCACTG
ATGCCTCCGTGTAAGGGGGATTTCTGTTCATGGGGGTAATGATACCGATGAAACGAGAGAG
GATGCTCACGATACGGGTTACTGATGATGAACATGCCCGGTTACTGGAACGTTGTGAGGGT
AAACAACTGGCGGTATGGATGCGGCGGGACCAGAGAAAAATCACTCAGGGTCAATGCCA
GCGCTTCGTTAATACAGATGTAGGTGTTCCACAGGGTAGCCAGCAGCATCCTGCGATGCAG
ATCCGGAACATAATGGTGCAGGGCGCTGACTTCCGCGTTTCCAGACTTTACGAAACACGGA
AACCGAAGACCATTCATGTTGTTGCTCAGGTCGCAGACGTTTTGCAGCAGCAGTCGCTTCA
CGTTCGCTCGCGTATCGGTGATTCATTCTGCTAACCAGTAAGGCAACCCCGCCAGCCTAGC
CGGGTCCTCAACGACAGGAGCACGATCATGCTAGTCATGCCCCGCGCCCACCGGAAGGAG
CTGACTGGGTTGAAGGCTCTCAAGGGCATCGGTCGAGATCCCGGTGCCTAATGAGTGAGC
TAACTTACATTAATTGCGTTGCGCTCACTGCCCGCTTTCCAGTCGGGAAACCTGTCGTGCCA
GCTGCATTAATGAATCGGCCAACGCGCGGGGAGAGGCGGTTTGCGTATTGGGCGCCAGGG
TGGTTTTTCTTTTCACCAGTGAGACGGGCAACAGCTGATTGCCCTTCACCGCCTGGCCCTGA
GAGAGTTGCAGCAAGCGGTCCACGCTGGTTTGCCCCAGCAGGCGAAAATCCTGTTTGATG
GTGGTTAACGGCGGGATATAACATGAGCTGTCTTCGGTATCGTCGTATCCCACTACCGAGA
TGTCCGCACCAACGCGCAGCCCGGACTCGGTAATGGCGCGCATTGCGCCCAGCGCCATCT
GATCGTTGGCAACCAGCATCGCAGTGGGAACGATGCCCTCATTCAGCATTTGCATGGTTTG

TTGAAAACCGGACATGGCACTCCAGTCGCCTTCCCGTTCCGCTATCGGCTGAATTTGATTGC
GAGTGAGATATTTATGCCAGCCAGCCAGACGCAGACGCGCCGAGACAGAACTTAATGGGC
CCGCTAACAGCGCGATTTGCTGGTGACCCAATGCGACCAGATGCTCCACGCCCAGTCGCG
TACCGTCTTCATGGGAGAAAATAATACTGTTGATGGGTGTCTGGTCAGAGACATCAAGAAA
TAACGCCGGAACATTAGTGCAGGCAGCTTCCACAGCAATGGCATCCTGGTCATCCAGCGG
ATAGTTAATGATCAGCCCACTGACGCGTTGCGCGAGAAGATTGTGCACCGCCGCTTTACAG
GCTTCGACGCCGCTTCGTTCTACCATCGACACCACCACGCTGGCACCCAGTTGATCGGCGC
GAGATTTAATCGCCGCGACAATTTGCGACGGCGCGTGCAGGGCCAGACTGGAGGTGGCAA
CGCCAATCAGCAACGACTGTTTGCCCGCCAGTTGTTGTGCCACGCGGTTGGGAATGTAATT
CAGCTCCGCCATCGCCGCTTCCACTTTTTCCCGCGTTTTCGCAGAAACGTGGCTGGCCTGGT
TCACCACGCGGGAAACGGTCTGATAAGAGACACCGGCATACTCTGCGACATCGTATAACG
TTACTGGTTTCACATTCACCACCCTGAATTGACTCTCTTCCGGGCGCTATCATGCCATACCG
CGAAAGGTTTTGCGCCATTCGATGGTGTCCGGGATCTCGACGCTCTCCCTTATGCGACTCCT
GCATTAGGAAGCAGCCCAGTAGTAGGTTGAGGCCGTTGAGCACCGCCGCCGCAAGGAATG
GTGCATGCAAGGAGATGGCGCCCAACAGTCCCCCGGCCACGGGGCCTGCCACCATACCC
ACGCCGAAACAAGCGCTCATGAGCCCGAAGTGGCGAGCCCGATCTTCCCCATCGGTGATG
TCGGCGATATAGGCGCCAGCAACCGCACCTGTGGCGCCGGTGATGCCGGCCACGATGCGT
CCGGCGTAGAGGATCGAGATCGATCTCGATCCCGCGAAATTAATACGACTCACTATA

Seq 4: pASPV4

GGGGAATTGTGAGCGGATAACAATTCCCCTCTAGAAATAATTTTGTTTAACTTTAAGAAGGAGAT
ATACCATGCATCATCACCACCACCACCACCATTCACATACCACTCCGTGGACCAATCCGGGTCT
GGCGGAGAACTTCATGAACTCCTTTATGCAGGGCCTGAGCAGCATGCCGGGTTTCACCGCAAG
CCAGCTGGACGACATGAGCACGATTGCGCAAAGCATGGTTCAGTCGATCCAGAGCTTGGCGGC
CCAGGGTCGCACCTCCCCGAATAAGCTGCAGGCACTGAACATGGCGTTCGCCAGCTCCATGGC
AGAGATCGCGGCGTCTGAAGAAGGTGGTGGTAGTTTGTCTACGAAAACCAGCTCTATCGCGTCT
GCTATGAGCAACGCATTCCTGCAGACCACTGGTGTGGTGAACCAGCCATTTATCAATGAAATCA
CCCAGCTCGTTAGCATGTTTGCACAAGCTGGCATGAATGATGTTAGCGCGGCTAGCTTGGGCTA
CGGCCCAGGGGGGGCTTCGGGGGCCGCAGCAGCGGCGGCTGCTGCCGACGGTGGCCGTGGG
GGGTTATACGGCCGGGGCCGCGGGCTGGGCGGTGATTCTGCAGCCGCTGCGGCCGCTGCGGA
TGGTGGGCGCGGGGGGTACGGTGGACGGGGCCTGGGTGGTGACTCTGGTGCCGCAGCTGCGG
CTGCAGCCGCTGACGGTGGCCGCGGTGGCTATGGTGGTCGGGGTTTAGGGGGCGATAGTGCAG
CGGCGGCTGCCGACGGCGGGCGGGGCGGTACTAGTGTTACCAGCGGCGGCTACGGTTACGGC
ACCTCCGCGGCGGCTGGTGCGGGCGTCGCAGCGGGTAGCTATGCCGGCGCGGTTAATCGTCTG
AGCTCAGCGGAGGCAGCGTCTCGTGTTTCCTCCAACATCGCCGCGATTGCCTCGGGCGGCGCA
AGCGCTTTACCGTCTGTTATTAGCAACATTTATAGCGGCGTCGTGGCATCGGGCGTGAGCTCTA
ACGAGGCCCTGATTCAGGCACTTTTGGAACTGCTGTCTGCCTTGGTGCACGTGCTGAGCTCAGC
TAGTATCGGTAACGTGAGCTCCGTGGGTGTCGACAGCACGCTGAATGTAGTTCAGGATTCCGTG
GGCCAATATGTTGGTAAAAAGTAAGGACTCGTCTACTAGCGCAGCTTAATTAACCTAGGCTGCT
GCCACCGCTGAGCAATAACTAGCATAACCCCTTGGGGCCTCTAAACGGGTCTTGAGGGGTTTTT
TGCTGAAAGGAGGAACTATATCCGGATTGGCGAATGGGACGCGCCCTGTAGCGGCGCATTAAG
CGCGGCGGGTGTGGTGGTTACGCGCAGCGTGACCGCTACACTTGCCAGCGCCCTAGCGCCCGC
TCCTTTCGCTTTCTTCCCTTCCTTTCTCGCCACGTTCGCCGGCTTTCCCCGTCAAGCTCTAAATCG
GGGGCTCCCTTTAGGGTTCCGATTTAGTGCTTTACGGCACCTCGACCCCAAAAAACTTGATTAG
GGTGATGGTTCACGTAGTGGGCCATCGCCCTGATAGACGGTTTTTCGCCCTTTGACGTTGGAGT
CCACGTTCTTTAATAGTGGACTCTTGTTCCAAACTGGAACAACACTCAACCCTATCTCGGTCTAT
TCTTTTGATTTATAAGGGATTTTGCCGATTTCGGCCTATTGGTTAAAAAATGAGCTGATTTAACAA
AAATTTAACGCGAATTTTAACAAAATATTAACGTTTACAATTTCTGGCGGCACGATGGCATGAGA
TTATCAAAAAGGATCTTCACCTAGATCCTTTTAAATTAAAAATGAAGTTTTAAATCAATCTAAAGT
ATATATGAGTAAACTTGGTCTGACAGTTACCAATGCTTAATCAGTGAGGCACCTATCTCAGCGAT
CTGTCTATTTCGTTCATCCATAGTTGCCTGACTCCCCGTCGTGTAGATAACTACGATACGGGAGG
GCTTACCATCTGGCCCCAGTGCTGCAATGATACCGCGAGACCCACGCTCACCGGCTCCAGATTT
ATCAGCAATAAACCAGCCAGCCGGAAGGGCCGAGCGCAGAAGTGGTCCTGCAACTTTATCCGC
CTCCATCCAGTCTATTAATTGTTGCCGGGAAGCTAGAGTAAGTAGTTCGCCAGTTAATAGTTTGC
GCAACGTTGTTGCCATTGCTACAGGCATCGTGGTGTCACGCTCGTCGTTTGGTATGGCTTCATTC
AGCTCCGGTTCCCAACGATCAAGGCGAGTTACATGATCCCCCATGTTGTGCAAAAAAGCGGTTA
GCTCCTTCGGTCCTCCGATCGTTGTCAGAAGTAAGTTGGCCGCAGTGTTATCACTCATGGTTATG
GCAGCACTGCATAATTCTCTTACTGTCATGCCATCCGTAAGATGCTTTTCTGTGACTGGTGAGTA
CTCAACCAAGTCATTCTGAGAATAGTGTATGCGGCGACCGAGTTGCTCTTGCCCGGCGTCAATA
CGGGATAATACCGCGCCACATAGCAGAACTTTAAAAGTGCTCATCATTGGAAAACGTTCTTCGG
GGCGAAAACTCTCAAGGATCTTACCGCTGTTGAGATCCAGTTCGATGTAACCCACTCGTGCACC
CAACTGATCTTCAGCATCTTTTACTTTCACCAGCGTTTCTGGGTGAGCAAAAACAGGAAGGCAA
AATGCCGCAAAAAAGGGAATAAGGGCGACACGGAAATGTTGAATACTCATACTCTTCCTTTTTC

AATCATGATTGAAGCATTTATCAGGGTTATTGTCTCATGAGCGGATACATATTTGAATGTATTTAG
AAAAATAAACAAATAGGTCATGACCAAAATCCCTTAACGTGAGTTTTCGTTCCACTGAGCGTCA
GACCCCGTAGAAAAGATCAAAGGATCTTCTTGAGATCCTTTTTTTCTGCGCGTAATCTGCTGCTT
GCAAACAAAAAAACCACCGCTACCAGCGGTGGTTTGTTTGCCGGATCAAGAGCTACCAACTCTT
TTTCCGAAGGTAACTGGCTTCAGCAGAGCGCAGATACCAAATACTGTCCTTCTAGTGTAGCCGT
AGTTAGGCCACCACTTCAAGAACTCTGTAGCACCGCCTACATACCTCGCTCTGCTAATCCTGTTA
CCAGTGGCTGCTGCCAGTGGCGATAAGTCGTGTCTTACCGGGTTGGACTCAAGACGATAGTTAC
CGGATAAGGCGCAGCGGTCGGGCTGAACGGGGGGTTCGTGCACACAGCCCAGCTTGGAGCGA
ACGACCTACACCGAACTGAGATACCTACAGCGTGAGCTATGAGAAAGCGCCACGCTTCCCGAA
GGGAGAAAGGCGGACAGGTATCCGGTAAGCGGCAGGGTCGGAACAGGAGAGCGCACGAGGG
AGCTTCCAGGGGGAAACGCCTGGTATCTTTATAGTCCTGTCGGGTTTCGCCACCTCTGACTTGA
GCGTCGATTTTTGTGATGCTCGTCAGGGGGGCGGAGCCTATGGAAAAACGCCAGCAACGCGGC
CTTTTTACGGTTCCTGGCCTTTTGCTGGCCTTTTGCTCACATGTTCTTTCCTGCGTTATCCCCTGAT
TCTGTGGATAACCGTATTACCGCCTTTGAGTGAGCTGATACCGCTCGCCGCAGCCGAACGACCG
AGCGCAGCGAGTCAGTGAGCGAGGAAGCGGAAGAGCGCCTGATGCGGTATTTTCTCCTTACGC
ATCTGTGCGGTATTTCACACCGCATATATGGTGCACTCTCAGTACAATCTGCTCTGATGCCGCAT
AGTTAAGCCAGTATACACTCCGCTATCGCTACGTGACTGGGTCATGGCTGCGCCCCGACACCCG
CCAACACCCGCTGACGCGCCCTGACGGGCTTGTCTGCTCCCGGCATCCGCTTACAGACAAGCT
GTGACCGTCTCCGGGAGCTGCATGTGTCAGAGGTTTTCACCGTCATCACCGAAACGCGCGAGG
CAGCTGCGGTAAAGCTCATCAGCGTGGTCGTGAAGCGATTCACAGATGTCTGCCTGTTCATCCG
CGTCCAGCTCGTTGAGTTTCTCCAGAAGCGTTAATGTCTGGCTTCTGATAAAGCGGGCCATGTTA
AGGGCGGTTTTTTCCTGTTTGGTCACTGATGCCTCCGTGTAAGGGGGATTTCTGTTCATGGGGGT
AATGATACCGATGAAACGAGAGAGGATGCTCACGATACGGGTTACTGATGATGAACATGCCCG
GTTACTGGAACGTTGTGAGGGTAAACAACTGGCGGTATGGATGCGGCGGGACCAGAGAAAAAT
CACTCAGGGTCAATGCCAGCGCTTCGTTAATACAGATGTAGGTGTTCCACAGGGTAGCCAGCAG
CATCCTGCGATGCAGATCCGGAACATAATGGTGCAGGGCGCTGACTTCCGCGTTTCCAGACTTT
ACGAAACACGGAAACCGAAGACCATTCATGTTGTTGCTCAGGTCGCAGACGTTTTGCAGCAGC
AGTCGCTTCACGTTCGCTCGCGTATCGGTGATTCATTCTGCTAACCAGTAAGGCAACCCCGCCA
GCCTAGCCGGGTCCTCAACGACAGGAGCACGATCATGCTAGTCATGCCCCGCGCCCACCGGAA
GGAGCTGACTGGGTTGAAGGCTCTCAAGGGCATCGGTCGAGATCCCGGTGCCTAATGAGTGAG
CTAACTTACATTAATTGCGTTGCGCTCACTGCCCGCTTTCCAGTCGGGAAACCTGTCGTGCCAGC
TGCATTAATGAATCGGCCAACGCGCGGGGAGAGGCGGTTTGCGTATTGGGCGCCAGGGTGGTT
TTTCTTTTCACCAGTGAGACGGGCAACAGCTGATTGCCCTTCACCGCCTGGCCCTGAGAGAGTT
GCAGCAAGCGGTCCACGCTGGTTTGCCCCAGCAGGCGAAAATCCTGTTTGATGGTGGTTAACG
GCGGGATATAACATGAGCTGTCTTCGGTATCGTCGTATCCCACTACCGAGATGTCCGCACCAAC
GCGCAGCCCGGACTCGGTAATGGCGCGCATTGCGCCCAGCGCCATCTGATCGTTGGCAACCAG
CATCGCAGTGGGAACGATGCCCTCATTCAGCATTTGCATGGTTTGTTGAAAACCGGACATGGCA
CTCCAGTCGCCTTCCCGTTCCGCTATCGGCTGAATTTGATTGCGAGTGAGATATTTATGCCAGCC
AGCCAGACGCAGACGCGCCGAGACAGAACTTAATGGGCCCGCTAACAGCGCGATTTGCTGGTG
ACCCAATGCGACCAGATGCTCCACGCCCAGTCGCGTACCGTCTTCATGGGAGAAAATAATACTG
TTGATGGGTGTCTGGTCAGAGACATCAAGAAATAACGCCGGAACATTAGTGCAGGCAGCTTCC
ACAGCAATGGCATCCTGGTCATCCAGCGGATAGTTAATGATCAGCCCACTGACGCGTTGCGCG
AGAAGATTGTGCACCGCCGCTTTACAGGCTTCGACGCCGCTTCGTTCTACCATCGACACCACCA
CGCTGGCACCCAGTTGATCGGCGCGAGATTTAATCGCCGCGACAATTTGCGACGGCGCGTGCA

GGGCCAGACTGGAGGTGGCAACGCCAATCAGCAACGACTGTTTGCCCGCCAGTTGTTGTGCCA
CGCGGTTGGGAATGTAATTCAGCTCCGCCATCGCCGCTTCCACTTTTTCCCGCGTTTTCGCAGAA
ACGTGGCTGGCCTGGTTCACCACGCGGGAAACGGTCTGATAAGAGACACCGGCATACTCTGCG
ACATCGTATAACGTTACTGGTTTCACATTCACCACCCTGAATTGACTCTCTTCCGGGCGCTATCA
TGCCATACCGCGAAAGGTTTTGCGCCATTCGATGGTGTCCGGGATCTCGACGCTCTCCCTTATG
CGACTCCTGCATTAGGAAGCAGCCCAGTAGTAGGTTGAGGCCGTTGAGCACCGCCGCCGCAAG
GAATGGTGCATGCAAGGAGATGGCGCCCAACAGTCCCCCGGCCACGGGGCCTGCCACCATAC
CCACGCCGAAACAAGCGCTCATGAGCCCGAAGTGGCGAGCCCGATCTTCCCCATCGGTGATGT
CGGCGATATAGGCGCCAGCAACCGCACCTGTGGCGCCGGTGATGCCGGCCACGATGCGTCCG
GCGTAGAGGATCGAGATCGATCTCGATCCCGCGAAATTAATACGACTCACTATA
